# STatin TReatment for COVID-19 to Optimise NeuroloGical recovERy (STRONGER): study protocol for a randomised, open label clinical trial in patients with persistent neurological symptoms after COVID-19 infection

**DOI:** 10.1136/bmjopen-2024-089382

**Published:** 2025-04-14

**Authors:** Carlos Delfino, Cheryl Carcel, Xiaolei Lin, Paula Muñoz-Venturelli, Sharon L Naismith, Mark Woodward, Ruth Peters, Nirupama Wijesuriya, Meng Law, Ian H Harding, Xia Wang, Julian Elliott, Karin Leder, Owen Hutchings, Ximena Stecher, Sophia Zoungas, Craig S Anderson

**Affiliations:** 1 Centro de Estudios Clínicos, Instituto de Ciencias e Innovación en Medicina (ICIM), Universidad del Desarrollo Facultad de Medicina Clínica Alemana, Las Condes, Chile; 2 Faculty of Medicine, University of New South Wales, The George Institute for Global Health, Sydney, New South Wales, Australia; 3 Data science, Fudan University, Shanghai, China; 4 Charles Perkins Centre and The University of Sydney, The University of Sydney Brain and Mind Centre, Camperdown, New South Wales, Australia; 5 School of Public Health, Imperial College London, The George Institute for Global Health UK, Oxford, Oxfordshire, UK; 6 Department of Neuroscience, School of Translational Medicine, Monash University, Melbourne, Victoria, Australia; 7 Department of Epidemiology and Preventive Medicine, School of Public Health and Preventive Medicine, Monash University, Melbourne, Victoria, Australia; 8 Royal Prince Alfred Hospital, Camperdown, New South Wales, Australia; 9 Departamento de Imagenología, Clínica Alemana de Santiago, Facultad de Medicina Clínica Alemana Universidad del Desarrollo, Clinica Alemana de Santiago SA, Vitacura, Metropolitan Region, Chile; 10 Institute of Science and Technology for Brain-inspired Intelligence, Fudan University, Shanghai, Shanghai, China

**Keywords:** Protocols & guidelines, COVID-19, Post-Acute COVID-19 Syndrome, Clinical Protocols

## Abstract

**Introduction:**

Increasing awareness of the high frequency, wide spectrum and disabling nature of symptoms that can persist following COVID-19 infection has prompted the investigation of management strategies. Our study aims to determine the effectiveness of atorvastatin on cognitive function, physical activity, mood, health-related quality of life and features of neurovascular impairment and neuroinflammation in adults with ongoing neurological symptoms after COVID-19 infection.

**Methods and analysis:**

The STatin TReatment for COVID-19 to Optimise NeuroloGical recovERy study is an ongoing international, investigator-initiated and conducted, multicentre, prospective, randomised, open label, blinded endpoint trial with fixed time points for outcome assessments. A total of 410 participants with long covid neurological symptoms were planned to be randomly assigned to either the intervention group to receive 40 mg atorvastatin for 12 months or to a control group of no treatment, on top of usual care.

**Ethics and dissemination:**

This study protocol was designed, implemented and reported, in accordance with the International Conference on Harmonisation guidelines for Good Clinical Practice, the National Health and Medical Research Council of Australia, the National Statement on Ethical Conduct in Human Research and with the ethical principles laid down in the World Medical Association Declaration of Helsinki. Central ethics committee approval was obtained from Sydney Local Health District Royal Prince Alfred Hospital Ethics (No: X21-0113 and 2021/ETH00777 10) in Australia. Site-specific ethics committee approvals were obtained elsewhere before any local study activities. All participants provided written informed consent.

**Trial registration number:**

The study protocol is registered at Clinicaltrials.gov (NCT04904536).

**STRENGTHS AND LIMITATIONS OF THIS STUDY**

- This study was designed as a prospective, randomised, open label, blinded endpoint trial, comparing atorvastatin 40 mg to standard care for the treatment of neurological symptoms associated with long covid.
- The study will be able to systematically and comprehensively characterise patients with neurological long COVID.
- The study is open-label, but cross-over to the use of atorvastatin is unlikely, and the primary outcome measure is objective.

## Introduction

 The epidemiological pattern and clinical spectrum, pathogenesis and complications in people infected with SARS-CoV-2 have been well-described.^1 2^ However, it is increasingly recognised that many patients have persistent symptoms, in particular, related to cardiorespiratory, mobility, cognitive and psychological function.^3^,^5^ This phenomenon has been termed ‘long COVID’ to describe symptoms that continue or develop after acute COVID-19, arbitrarily defined as either ongoing symptomatic COVID-19 (from 4 weeks to 12 weeks) or post-COVID-19 syndrome (12 weeks or more).^6^

As many as three-quarters of patients have at least one ongoing symptom several months after onset of the infection.^7^ About 5–10% of individuals experience neurological symptoms, including issues with higher level cognitive functions such as sustained attention, cognitive flexibility and memory, collectively termed ‘brain fog’, along with symptoms such as headaches, dizziness, anxiety, depression, insomnia and fatigue.^8^,^11^ Such manifestations may persist even among individuals with mild or absent respiratory symptoms.^12^ These late neurocognitive problems following COVID-19 could be attributed to: (1) direct non-resolving low-grade inflammation or immune reactions in the brain^13^ and (2) indirect cerebral injury from hypotension, hypoxia and metabolic dysfunction from effects on the heart, lungs and other organs.^4 14^ Full neurocognitive recovery post-COVID-19 may take months,^11^ and akin to other forms of brain injury, it may have long-term impacts on brain health and neurodegenerative conditions such as Alzheimer’s disease.^15^

Several studies have used specific tests to diagnose neurocognitive symptoms in long covid patients, revealing impairments in executive functions, attention and memory.^16^ These deficits manifest clinically as difficulties in planning, sustaining focus, recalling information and effectively processing new stimuli, which significantly impact daily functioning and quality of life. Such neurocognitive impairments may be linked to underlying neuropathological changes elucidated through advanced imaging modalities and biomarker analyses.

MRI findings in the brain, particularly of free water quantification using diffusion-weighted sequences, are a sensitive marker of small vessel disease,^17^ cognitive impairment (especially decision-making performance and working memory)^18^ and neurodegenerative disease.^19^ Increases in brain free water reflect an enlargement of extracellular spaces within the cellular matrix of the grey matter or axonal pathways of the white matter due to vasogenic oedema, neuroinflammatory gliosis and/or loss of neuropil or myelin.^20^ While these changes are traditionally evaluated through histopathological analyses, advanced MRI techniques provide a valuable non-invasive approach to approximating pathological processes.

Additionally, blood markers of endothelial damage, immune function (cytokines/chemokines and acute phase proteins) and neuronal and glial health (eg, neurofilament light chain and glial fibrillar acidic protein) in the acute phase of COVID-19 may function as predictors of long covid.^21^ These insights underscore the multifactorial nature of neurocognitive symptoms in long covid and the importance of integrating clinical, imaging and biomarker data to better understand and manage this condition.

Statins, widely prescribed for cardiovascular disease prevention, inhibit cholesterol biosynthesis by targeting 3-hydroxy-3-methylglutaryl coenzyme A reductase. They also have anti-inflammatory, antioxidant, vascular endothelial function-enhancing, plaque-stabilising and platelet aggregation-inhibiting effects.^22^,^24^ Statins may have neuroprotective effects beyond their cholesterol-lowering properties.^25^ Epidemiological associations show a reduced risk of all-cause dementia and Alzheimer’s disease in individuals using statins,^1526^,^29^ with effects likely related to type, dose, duration and onset timing over the life course.^30 31^ Given the key role of the neurovascular unit in modifying the brain’s susceptibility to injury, any benefit of statin therapy is more likely when initiated promptly after an acute inflammatory insult. As atorvastatin crosses the blood-brain barrier due to its high lipophilicity,^32^ it shows promise as a treatment of neurological symptoms of prolonged COVID-19.^33^

Here, we outline the protocol for the STatin TReatment for COVID-19 to Optimise NeuroloGical recovERy (STRONGER) study, which aims to determine the efficacy of atorvastatin in primarily mitigating cognitive decline by reducing (or stabilising) neuroinflammation indicated by MRI changes and blood biomarkers.

### Objectives

To determine the effects of a standard 40 mg dose of atorvastatin on improving neurological outcomes in adults experiencing persistent cognitive symptoms after acute infection with COVID-19. The primary outcome is cognitive processing speed, assessed by the symbol digit modalities test (SDMT); the key secondary outcome is total white matter free water on MRI. Additionally, the study will investigate other cognitive measures and health assessments and MRI markers, including white matter hyperintensity volume, perivascular space enlargement, grey matter cortical thickness, white matter microstructure, basal ganglia iron load and cerebral perfusion. A cost-effectiveness analysis will also be undertaken.

## Methods and analysis

### Study design and setting

STRONGER is an international, investigator-initiated and conducted, multicentre, prospective, randomised, open label, blinded endpoint study, with a fixed time point for outcome assessments. Open label indicates that participants and researchers are aware of the treatment allocation. Participants are centrally randomised (1:1) to either the intervention group to receive 40 mg atorvastatin for 12 months on top of usual care or to the control group to receive usual care. Recruitment began on 5 May 2022 and ended in July 2024. The follow-up of participants is until July 2025, and the results are planned to be presented in October 2025.

The study was approved for conduct at three medical clinic sites: the Brain and Mind Centre of the University of Sydney, Monash University in Australia (central ethics approval from Sydney Local Health District Royal Prince Alfred Hospital Ethics, numbers X21-0113 and 2021/ETH00777 10, date of first approval 19 May 2021) and at the Clínica Alemana Universidad del Desarrollo, Santiago, Chile (Comité ético científico: Facultad de Medicina, Clínica Alemana de Santiago, Universidad del Desarrollo, number 2021–75, date of first approval 20 August 2021; government approval: number EC1707587, date: 17 November 2021) as outlined in online supplemental file 1. All subjects provided written informed consent according to the forms shown in online supplemental file 2.

### Eligibility of study participants

Eligible participants are aged ≥18 years, have a history of COVID-19 that is confirmed by a positive PCR test, a rapid antigen test or as per local guidelines for COVID-19 diagnosis at the time of screening and have ongoing neurological symptoms as a result of COVID-19. These symptoms included problems with memory, concentration, sleep disturbance and fatigue and were systematically identified through administration of the Somatic and Psychological Health Report-34 item questionnaire^34^ and of any reported loss of smell (anosmia). They must be able to participate in all procedures and provide written informed consent.

Participants were excluded if they had any of the following: evidence of dementia and/or significant cognitive impairment on screening; a severe comorbid medical (eg, renal failure) or psychiatric condition (ie, drug or alcohol dependence and schizophrenia) that prevented participation; history of traumatic brain injury with loss of consciousness (>30 min) within the last 2 years; ongoing long-term use or clear indication or contraindication for statin use; evidence of severe or significant liver disease; creatine kinase levels more than twice the upper limit of normal; being female of childbearing potential and unable or unwilling to use a reliable method of contraception, currently breastfeeding or planned pregnancy. For the subgroup of participants who agree to undergo MRI, they must have had no contraindication due to metallic body parts or claustrophobia. Finally, participants were excluded if their medical history might, in the opinion of their treating physician, put them at significant risk if they were to participate in the trial.

### Intervention

**Figure 1:**
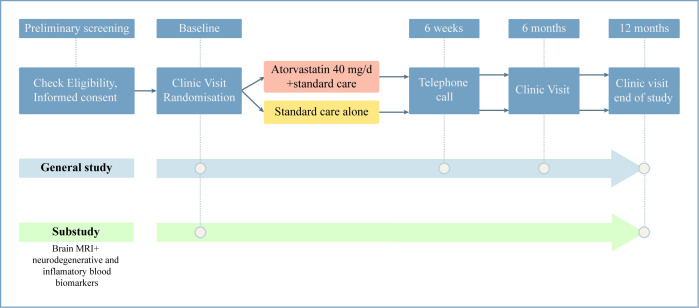
Flow chart of study design.

Participants who meet the eligibility criteria were randomised to receive standard care or atorvastatin 40 mg on top of standard care for a period of 12 months (figure 1). The randomisation was stratified by country, time (<6 vs ≥6 months since acute COVID-19 illness), age (<60 vs ≥60 years), current anosmia (yes vs no) that has occurred with temporal relation to COVID-19 and participation in the MRI/biomarker substudy. The randomisation allocation was blinded to researchers conducting the cognitive assessments and endpoint adjudication, and participants, physicians and other study team members were aware of the treatment allocation.

### Visit summary

The study incorporates a blend of inperson visits and telephone interviews (figure 1). Preliminary screening was via telephone or teleconference to obtain informed consent and for review of the inclusion and exclusion criteria. Inperson visits encompass baseline assessments, a clinical evaluation at 6 months and an end-of-study assessment at 12 months. During the baseline visit, various evaluations were performed, including fasting blood tests, clinical assessment, cognitive tests, health questionnaires and physical activity assessments (table 1). Randomisation was undertaken after these assessments were completed, with the participants only being informed of their allocation when all the baseline evaluations were finished, to mitigate the risk of differential withdrawal between the study arms. Furthermore, participants who were enrolled in the substudy are required to undergo MRI scans and blood biomarker analyses at baseline and the end of follow-up. The 6-month visit focused on clinical evaluation and adverse event assessment within the treatment group. On the 12-month follow-up, the baseline assessments are repeated.

**Table 1:** Study outcomes

Name		Purpose	Assessment
Primary outcome			
	Symbol Digit Modalities Test^35^	Assesses frontal lobe executive processing	Requires participants to match numbers to symbols according to a key, containing nine abstract symbols, each paired with a number. Performance is measured by the number of correct symbols within 90 s.
Secondary outcomes			
Neuropsychological assessments	Rey Auditory Verbal Learning test^40^	Assesses memory and learning	Requires participants to learn a 15-item word list over five learning trials, followed by a distractor list and short and long-delayed recall.
	Oral Trail Making Test A and B^41^	Summarises visual search, scanning, speed of processing, mental flexibility and executive functions	Has two parts: part A—participants count from 1 to 25 as quickly as possible. In Part B—participants alternately count numbers up to 13 and recite letters of the alphabet up to L as quickly as possible.The primary performance metric is the time in seconds required to complete each of the two parts of the test.
	Delis-Kaplan Executive Functioning System ‘Stroop’ Colour-Word Interference Test^42^	Measure executive functions	Has four parts: colour naming, word reading, inhibition and inhibition/switching.Performance is measured by completion time for each trial.Composite score: colour naming and word reading times.Contrast scores: inhibition versus colour naming, inhibition/switching versus combined colour naming and word reading and inhibition/switching versus inhibition, assess disproportionate impairments in higher-level functions compared with component functions.
	Semantic Fluency^43 44^	Assesses language processing	Requires the generation of as many ‘animal names’ in 1 min. Performance is measured by counting the number of correct unique semantic category items produced.
Health assessments	COVID-19 Yorkshire Rehabilitation Scale^45^	Evaluate the long-term impact of COVID- 19	It is administered by self-report or clinician. It will be used as a baseline measure and then at follow-up at 18 months as an assessment of response to therapy.
	Patient Health Questionnaire^46^	Measure the presence and severity of depression	Evaluates each of the nine DSM-V major depression criteria on a scale from ‘0’ (not at all) to ‘3’ (nearly every day). It aids in making a preliminary diagnosis of depression in at-risk populations (eg, individuals with coronary heart disease or after stroke), with scores ≥10 indicating probable clinically significant depression.
	General Anxiety Disorder^47^	Measure the presence and severity of generalised anxiety	A score ≥10 represents a reasonable cut point for identifying cases of generalised anxiety. Cut points of 5, 10 and 15 might be interpreted as representing mild, moderate and severe levels of anxiety.
	Pittsburgh Sleep Quality Index^48^	Assesses overall sleep quality in clinical populations	Covers 19 self-reported items across seven subcategories: subjective sleep quality, sleep latency, sleep duration, habitual sleep efficiency, sleep disturbances, use of sleeping medication and daytime dysfunction; with five additional questions rated by the respondent’s roommate or bed partner, if available. Scores >5 are generally indicative of poor sleep quality.
	EQ-5D-5L^49^	Measures health-related quality of life	Across five dimensions (mobility, self-care, usual activities, pain/discomfort and anxiety/depression), each rated according to five levels, as well as providing an integrated utility score for calculating an overall score against population-based preference weights.
	International Physical Activity Questionnaire-long form^50^	Physical activity assessment	A valid 27-item self-reported assessment to provide an estimate of physical activity and sedentary behaviour for adults aged 15–69 years. Participants reflect on activities over the previous 7 days across five domains: (1) occupational physical activity; (2) transportation physical activity; (3) housework, house maintenance and caring for family; (4) recreation, sport and leisure-time physical activity; (5) time spent sitting. Physical activity scores can be calculated in either categorical score (high, medium and low) or MET minutes per week.
Substudy outcomes			
MRI	Total white matter free water (diffusion MRI)		Using multishell diffusion spectral imaging with fibre orientation and compartment modelling.
	Others		MRI markers of white matter hyperintensity volume, enlarged perivascular space volume, total grey matter volume, white matter microstructure (fractional anisotropy), basal ganglia iron load and total cerebral perfusion.
Blood biomarkers	Neurodegenerative markers		Ptau-181, neurofilament light chain, Aβ42/40 and DNA extraction for apolipoprotein E genotype.
	Peripheral markers		Interleukin (IL)-6, IL-1β, NAD+, TNF-α, hsCRP

DSM-V denotes Diagnostic and Statistical Manual of Mental Disorders, Fifth Edition; EQ-5D-5L EuroQoL Group 5-Dimension 5-level self-report questionnaire ; hs-CRP high-sensitivity C-reactive protein; MET metabolic equivalent (1 MET is the energy spent sitting at rest); NAD nicotinamide adenine dinucleotide; TNF tumour necrosis factor.

Telephone interviews complement the inperson visits, particularly at the 6-week mark, to evaluate adverse events, medication adherence and changes in concomitant medication. Every effort is made to conduct assessments remotely for participants unable to attend inperson visits.

### Outcomes

Primary outcome is processing speed, assessed by the SDMT,^35 36^ which evaluates frontal lobe executive processing. Participants match numbers to symbols based on a cue of 9 abstract symbols paired with numbers. Performance is measured by the number of correct symbols within 90 s.

Secondary outcomes include a comprehensive battery of assessments covering executive functions, memory, processing speed and other domains, alongside evaluations of health status, MRI and blood biomarkers. A cost-effectiveness analysis, relative to standard care, will be executed. The table provides a detailed overview of all evaluations.

Cognitive assessments are administered by trained research psychologists specialised in cognitive measures. Importantly, these assessments can be conducted either in person or via videoconference.

### Safety outcomes

This study documents expected adverse reactions to the study medication of special interest (AESI), including myalgia, nausea, elevated blood glucose, elevated creatine kinase, abdominal pain, new-onset diabetes mellitus and rhabdomyolysis. These AESIs are closely monitored at specific visits to assess participants’ tolerability to study drugs, noting whether they are new or continuing from baseline, regardless of severity. Additionally, all serious adverse events (SAEs)^37^ and adverse events (AEs) are reported. If treatment is discontinued as a result of any AE, serious or not, the site study team is to provide details of all events that led to the discontinuation of treatment.

### Sample size

A sample of 410 patients was estimated to provide 80% power (α=0.05) to detect at least a 0.3 SD effect size difference between groups, assuming equal group participation. These calculations assume a modest 5% non-compliance and 5% dropout over 12 months of follow-up. The SDMT age-adjusted mean score is estimated at 60 (SD 13) at baseline (based on healthy control data).^35^ The effect is based on trials of the use of statins for the prevention of dementia and trials of treatments for multiple sclerosis, where achieved effect sizes of 0.3–0.4 are clinically meaningful and likely to confer public health benefits.^38^ For the substudy of participants undergoing MRI, a sample size of 220 (110 per group) is estimated to provide 80% power (α=0.05) to detect an effect size of relative difference of 5.0–6.5 (variance between groups/variance within groups) for the imaging endpoints, assuming a 20% drop out. The study statistician (XW) used the software package PASS V.15 (NCSS, Kaysville, Utah, USA; http://www.ncss.com/software/pass) for these power calculations.

### Data collection methods

All data entry is completed via a secure web-based data management system (IBM Clinical Development) at The George Institute for Global Health. Data entry is performed at the participating sites by authorised site staff who have completed training and been given appropriate role-based access to the system. Data logic and consistency checks are programmed into the data entry forms so that data entry errors are captured in real-time and queries are auto-generated. Authorised electronic signatures are used to lock completed data entry forms once all data queries have been resolved within the system. Data entry and all subsequent changes or deletions are captured in an accessible audit trail. Coding of outcomes is centrally performed either automatically via the IBM coding module or manually by the Central Coordinating Centre (CCC). All coding is reviewed and verified by a medical monitor. Reporting within the system is used for regular data reviews and overall trial monitoring. Data are stored and backed up on the IBM cloud servers in the USA.

### Statistical analysis plan

The study will follow the intention-to-treat principle for analysis. Baseline characteristics will be summarised by treatment group. Continuous baseline characteristics will be described by means (SD), if approximately normally distributed, or by medians (IQR), if normality cannot be assumed. Categorical baseline characteristics will be presented by frequency per category. Statistical comparisons of baseline characteristics between treatment and control groups are not planned.

The primary endpoint, SDMT score, will be summarised by means (SD) if normally distributed, or by medians (IQR) if normality cannot be assumed. The primary analysis will be conducted using linear regression, with the dependent variable being SDMT and the independent variable being group allocation (treatment vs control) with adjustment for the covariates of age, sex and site. A sensitivity analysis will be undertaken with adjustment for any meaningful baseline imbalances and clinically confounders. Another sensitivity analysis will be conducted by dichotomising SDMT according to population norms (average scores of 54 in Australia and 53.2 in Chile for people aged 44 years) and use of logistic regression models with adjustment for age, sex and study site.

Continuous secondary endpoints will be analysed using linear regression, adjusting for age, sex, study site, unbalanced baseline characteristics and clinically meaningful confounders. Binary secondary endpoints will be analysed using binary logistic regression, adjusting for age, sex, study site, unbalanced baseline characteristics and clinically meaningful confounders. Ordinal secondary endpoints with more than two categories will be analysed using a cumulative logit model, adjusting for age, sex, study site, unbalanced baseline characteristics and clinically meaningful confounders and with testing of the proportional odds assumption.

Descriptive statistics will be provided for safety data, where SAEs and treatment discontinuation will be tabulated using standard terminology. Heterogeneity of the treatment on the primary endpoint will be assessed in the predefined subgroups of age (<60 vs ≥60 years), time since COVID-19 diagnosis (<6 vs ≥6 months), baseline C-reactive protein levels (0–9 vs ≥10 mg/L), ethnicity (white Caucasian vs other) and prior cardiovascular risk (no vs yes for any of a history of hypertension, hyperlipidaemia, current smoker and body mass index (height (cm)/weight (kg) ≥10).^2^ The hypothesis is that there will be a larger relative treatment effect in younger people, in people with higher levels of inflammation by virtue of an earlier time from the acute COVID-19 infection, in people with raised inflammatory markers, in non-white individuals and those with elevated cardiovascular risk. A detailed outline of the statistical analyses will be specified a priori in a full statistical analysis plan prior to unblinding of the data. A modelled cost-utility analysis using trial data (health-related quality of life, captured by EuroQoL Group 5-Dimension 5-level self-report questionnaire; drug costs; health service utilisation costs, including AEs) will be conducted by comparing use of atorvastatin with standard of care. A 5-year time horizon will be undertaken, with analyses conducted in line with accepted Australian standards for use of economic evaluation in decision-making.

### Data monitoring

Trial data are monitored, using central risk-based monitoring principles, to detect any unusual patterns of data that would require further investigation. During the study, representatives of the CCC monitor site performance and quality via remote methods, including via videoconference, to ensure that the study is conducted in accordance with the protocol, International Conference on Harmonisation guidelines for Good Clinical Practice (ICH-GCP) guidelines and relevant ethical and regulatory requirements.

### Areas of potential bias

As this is a clinical trial, a major source of bias is *selection*, so we plan to compare the characteristics of participants with those in published cohorts and between those who were screened. Being an open trial, another main source of bias relates to *differential drop-out*, for example, participants in the control ‘no treatment’ group failing to return to follow-up assessments or withdrawing from the study. We will try to reduce this by ensuring that all participants fully understand the importance of participation and follow-up, irrespective of ceasing the study medication, and in maintaining good communication and relationships with participants. A final source of bias is *behavioural*, where participants in either group may change their lifestyle and attitude simply due to participation or in response to factors outside of the trial. We will monitor this by asking questions about use of concomitant medications, diet, smoking and exercise and through use of actigraphy as a proxy measure of sleep and physical activity. There is a low likelihood that the control ‘no treatment’ group will receive atorvastatin (ie, *treatment crossover*) as this can only be prescribed for people who are at high risk of cardiovascular disease.

### Patient and public involvement

The STRONGER study protocol was presented to the Brain Health Consumer Panel of The George Institute for Global Health at meetings in March and November of 2021. This panel comprises a dozen people with lived experience of brain health conditions or their caregivers. Feedback was sought regarding the protocol and patient-facing documents. Those with lived experience provided comments on the feasibility of the study, mode of recruitment, activities and information dissemination. This feedback was incorporated into the study where appropriate, and there was agreement to ensure that the findings of the study, along with a plain language summary, will be disseminated to participants of the trial after the study is published.

## Ethics and dissemination

This study protocol was designed and shall be implemented and reported in accordance with the ICH-GCP, the National Health and Medical Research Council, the National Statement on Ethical Conduct in Human Research and with the ethical principles laid down in the World Medical Associations Declaration of Helsinki. Potentially eligible participants are provided with information about the study, and informed consent is obtained from all participants prior to screening assessments.

Writing committees, with oversight by the steering committee, will be formed from members of the various committees, statisticians, research fellows and investigators. Authors of publications must meet the International Committee of Medical Journal Editors^39^ guidelines for authorship. The study has been approved by relevant ethics committees and regulatory bodies at country level and local sites in Australia and Chile. The current protocol is V.9.0, and all protocol updates have been approved by the Steering Committee and Ethics Committees and communicated with investigators and Data and Safety Monitoring Board members.

## Supplementary material

10.1136/bmjopen-2024-089382 online supplemental file 1

10.1136/bmjopen-2024-089382 online supplemental file 2
