# Supplementary material for: STatin TReatment for COVID-19 to Optimise NeuroloGical recovERy (STRONGER): study protocol for a randomised, open label clinical trial in patients with persistent neurological symptoms after COVID-19 infection
Source: BMJ Open. 2025 Apr 14;15(4):e089382. doi: 10.1136/bmjopen-2024-089382 (PMC11997840; doi:10.1136/bmjopen-2024-089382)
Supplement: online supplemental file 2 [file bmjopen-15-4-s002.pdf]

Master Participant Information Sheet/Consent Form

**Title**	Statin TReatment for COVID-19 to Optimise NeuroloGical recovERy
**Short Title**	STRONGER
**Protocol Number**	N/A
**Study Sponsor**	The George Institute for Global Health (TGI), University of New South Wales, Sydney, Australia
**Principal Investigator**	______________________ *[insert name]*

1. Introduction

You are invited to take part in a research study called STRONGER. This study aims to determine the effect of using a statin (a cholesterol-lowering medication) for improving the effects of "long COVID" symptoms on the brain. The treatment in this study involves the use of a commonly used statin called atorvastatin. The reason you have been invited to take part in this study is because you previously had COVID-19 and have some ongoing neurological symptoms (such as problems with your memory, concentration, mood, sleep disturbance and/or fatigue).

The study will be conducted by a group of international researchers and will involve 410 participants in Australia (mainly from the states of Victoria and New South Wales), Chile in South America, Hong Kong and possibly other countries. This study has received its main source of funding from the National Health and Medical Research Council (NHMRC) of Australia as well as the Medical Research Futures Fund (MRFF) and is being coordinated internationally by The George Institute for Global Health of the University of New South Wales, Australia.

This Participant Information Sheet and Consent Form tells you about the research study; explaining the tests and treatments that are involved. Knowing what is involved will help you decide if you want to take part.

Please read this information carefully. Ask questions about anything that you don't understand or want to know more about. Before deciding whether or not to take part, you might want to talk about it with a relative, friend, or your doctor.

Participation in this research is voluntary. If you don't wish to take part, you don't have to. You will receive the best possible care whether or not you decide to take part. Whatever your decision, please be assured that it will not affect your medical treatment, or your relationship with the staff who are caring for you.

Sometimes during the course of a study, new information becomes available about the treatment that is being studied. Whilst you are participating in this study, you will be kept informed of any significant new findings which may affect your willingness to continue in the study.

If you decide you want to take part in this research, you will be asked to sign the consent section of this form. By signing this form, you are telling us that you:

- understand what you have read,
- consent to take part in the research project,

- consent to have the tests and treatments that are described in this information sheet, and
- consent for the use of your personal and health information as described in the information sheet.

You will be given a copy of this Participant Information Sheet and Consent Form to keep.

2. What is the purpose of this research?

The purpose of this research is to investigate whether treatment with a statin (a cholesterol-lowering medication) on top of standard of care is better at improving COVID-19 related neurological symptoms when compared with standard care alone.

The medication being used in this trial is called atorvastatin. Atorvastatin is a statin, a class of drugs often prescribed by doctors to help lower cholesterol levels in the blood. By lowering these levels, they help prevent heart attacks and stroke. However, statins can also work by other mechanisms to reduce inflammation in the arteries and other parts of the body. Because atorvastatin is able to penetrate into the brain and reduce inflammation in the body, it may offer benefits in people who have had COVID-19 infection.

This medication is approved by different regulatory agencies, such as the Therapeutic Goods Administration (TGA) in Australia and the Food and Drug Administration (FDA) in the United States, for the treatment of people with elevated cholesterol. Essentially, the STRONGER study is investigating whether using a statin can help improve brain function and reduce the long-term effects caused by COVID-19. Atorvastatin has not yet been approved in Australia for the treatment of neurological symptoms caused by COVID-19. This study will be conducted under the Therapeutic Goods Administration (TGA) Clinical Trials Notification (CTN) Scheme. This allows the investigators to use this product for medical research purposes once the research has been assessed and approved by an authorised Human Research Ethics Committee (HREC).

COVID-19, an emerging infectious disease caused by severe acute respiratory syndrome coronavirus 2 (SARS-CoV-2), has affected nearly 100 million people globally and caused over 1.8 million deaths since the outbreak in China in late 2019. Symptoms and complications of those newly infected with COVID-19 have now been well described by scientists and those in the medical field, however, there is uncertainty over its long-term health consequences. There is increasing recognition that many patients have persistent symptoms, such as ongoing problems with breathing, energy, muscle strength, memory and mood. These symptoms are not confined to people who experienced a severe illness and were hospitalised with COVID-19. Studies have shown that up to three quarters of patients report at least one symptom several months after onset of the infection. The most common symptoms of 'long COVID' are fatigue, muscle weakness, disturbed sleep, and anxiety/depression. Approximately 5-10% of people report neurological symptoms, such as 'brain fog', headaches, poor concentration, and dizziness.

3. What does participation in this research involve?

Your duration of participation in this study is for 12 months.

Potentially eligible participants who express interest in the study will be contacted by the study coordinator to discuss the requirements of the study, briefly assess eligibility, and to share a copy of the PICF for the participant to review.

To confirm their eligibility, participants will progress through a 2-stage screening process involving:

Visit 1: a telehealth call and completion of online consent, and;

Visit 2: an in-person, more detailed clinical and cognitive assessment to confirm eligibility (this visit can be conducted via videoconference if required).

<u>Visit 1: Preliminary Screening – Telehealth</u>

You will be invited to attend a telehealth (via telephone or videoconference) appointment to complete the informed consent process and, after consent is provided, complete preliminary screening procedures. At this preliminary screening visit, a trained member of the study team, usually a doctor, nurse or research coordinator, will ask you a series of questions about your medical history and current medications. They will also complete a questionnaire with you and assess your memory and thinking which is called 'cognition' (this means the process of understanding how you remember things, problem solve, etc.).

The information collected about you at Visit 1 will determine if you are suitable to continue in the study. If you are suitable to continue in the study, you will be asked to attend a further screening visit.

Completing the preliminary screening process and signing the consent form does not guarantee your enrolment into the study. Rather, it allows the study team to commence discussions and investigations to assess your suitability to participate.

This visit will take approximately 30-45 minutes.

<u>Visit 2: Screening/Randomisation Visits (Clinic Visit)</u>

At visit 2 you will be invited to attend a morning appointment to a research clinic. You will be required to attend the visit in a <u>fasting state</u> (having not eaten anything for the previous 8 hours) to complete the screening process, and if eligible, for baseline assessments. You may be asked to provide a 20ml blood sample for laboratory measurements which will be collected at the clinic or at a local pathology collection centre. The blood tests required for this visit can also be collected prior to this visit at a pathology collection centre to allow for blood tests results to be available for this visit and/or to allow flexibility if ongoing visits will be done remotely.

At this screening visit, a trained member of the study team, usually a doctor, nurse or research coordinator, will ask you a series of questions about your medical history and current medications. You will also undergo a brief physical examination to collect clinical assessments that include the measurement of your blood pressure (BP) and heart rate (HR), your weight and height, as well as to assess your memory and thinking which is called 'cognition' (this means the process of understanding how you remember things, problem solve, etc.), assess your mood, general wellbeing, health-related questionnaires including quality of life and level of physical activity, and the health services that you have used recently.

At the end of this first visit, your study doctor or nurse will confirm that you are eligible to continue in the study. If you are eligible and you wish to continue to take part in the study, you will then be randomised (like 'tossing a coin') to receive study treatment. You will have a 50% chance of receiving:

- standard care, or
- standard care + atorvastatin 40mg

Both arms of the study will undergo the same assessments at each visit.

Sometimes we do not know which treatment is best for treating a condition. To find out, we need to compare different treatments. We put people into groups and give each group a different treatment. The results are compared to see if one is better. To try to make sure the groups are the same, each participant is put into a group by chance (randomised).

If you are randomised to the study medication arm, a member of the study team will mail out your study medication by courier (or you may collect it at a local pharmacy, if this is more appropriate). The pharmacy of Syntro Health will be supplying study medication and will be provided your contact details if you provide

consent to this study, in order to facilitate the delivery of the study medication. You will need to provide dispatch requirements i.e. either the authority to leave the medication at your home, whether a signature is required and to not leave at your home but re-deliver if you are not there. If re-delivery is attempted and you are not home again then it will be sent back to us. You will be instructed to take one tablet of study medication, once daily with or without food. It can be taken with your other tablets and at the same time every day. You will receive specific instructions about taking your study medication. If you miss a dose and it is within 12 hours of the usual time you take your medicine, then take it immediately. If it's more than 12 hours, continue taking your medicine the following day at the usual time with the usual dose. You will be asked to bring your remaining study medication with you to every clinic visit (at 6 and 12-month visits) for the study staff to check. A pre-paid envelope for Australia Post will be provided with the medication in case of any requirement at any point in the study to return the medication to us.

After this visit you will be mailed out an actigraphy watch which you will need to wear for a 7-day period in the next 2 weeks after this visit (post-randomisation). This 7-day period should include 2 weekend days and 5 week days i.e. Monday to Sunday. This watch will record your physical activity and sleep and will need to be returned promptly in the provided pre-paid return package to a central office for downloading of data.

Biomarker and MRI Sub-Study

You may be invited to participate in a sub-study for the collection and storage of blood for future neurodegenerative biomarker studies, and for a brain scan that uses magnets, called a magnetic resonance imaging (MRI), which will take approximately 45 mins to complete. You will be asked to sign a separate informed consent if you decide you would like to participate in this sub-study.

Altogether, this visit will take approximately 2-3 hours.

Visit 3: 6-Week Follow-up (Phone Visit)

During this phone visit, you will be required to answer some questions about your health and medications. You will be asked questions about your adherence to the study medication (if you are in the study medication arm) and to describe any side-effects that you may have had.

This visit will take approximately 15-30 minutes.

Visit 4: 6 -Month Follow-up Visit (Clinic Visit)

You will be asked questions about how well you tolerated the study medication (if you are in the study medication arm) and about any changes to your other medications. Your BP and HR will be measured. You will be asked to complete a few health-related questionnaires relating to your current level of physical activity and lifestyle. You will also be required to complete a few cognitive tests, some of which relate to your memory and mood. You will be asked some questions about your use of health services. Your returned study medication will be checked. You will be asked questions about your adherence to the study medication and to describe any side-effects you may have had. At this visit you will be provided with an actigraphy watch that you will need to wear for a 7-day period in the next 2 weeks after this visit (post-visit). This 7-day period should include 2 weekend days and 5 weekdays i.e. Monday to Sunday. This watch will record your physical activity and sleep, and will need to be returned promptly in the provided pre-paid return package to a central office so the data can be downloaded.

Following this visit, you will be provided with your next supply of study medication.

This visit will take approximately 2-3 hours.

Visit 5: 12 -Month Follow-up Visit (Clinic Visit)

The final follow-up visit will be conducted in the clinic and will involve a full set of assessments. You will undergo clinical assessments (BP, HR and weight) and answer some questions about your health and medications as well as complete some health-related questionnaires including your level of physical activity and cognitive tests (memory and mood). If you're in the study medication arm, your returned study medication will be checked, and you will be asked questions about your adherence to the study medication and to describe any side-effects you may have had. You will be asked to provide a 20 ml blood sample for laboratory measurement that requires prior fasting on the day. These samples will be collected at the clinic or at a local pathology collection centre.

At this visit you will be provided with an actigraphy watch which you will need to wear for a 7-day period in the next 2 weeks after this visit (post-visit). This 7-day period should include 2 weekend days and 5 weekdays i.e. Monday to Sunday. This watch will record your physical activity and sleep and will need to be returned promptly in the provided pre-paid return package to a central office so the data can be downloaded.

This visit will take approximately 3-4 hours to complete.

All study visits will be conducted at the same study clinic site. However, if there are circumstances that prevent you attending the clinic (e.g. pandemic), it may be possible for the clinic visits (at Visit 2, 4, and 5) to take place remotely via videoconference. If you are doing these visits remotely, you will be provided with a pathology request form and asked to attend your local pathology collection centre to have the blood samples collected. You will not be able to participate in the sub-study if visits are done entirely remotely.

With your permission, study medication will be provided to you via post or courier throughout the study.

4. What are the alternatives to participation?

Participation in this research study is voluntary. There may be other treatments available to manage your ongoing long COVID neurological symptoms. You can discuss these options with your general practitioner or medical specialist before you decide whether or not to take part in this research study.

5. Are there any benefits?

This study aims to generate further medical knowledge and to improve the management of patients with long COVID neurological symptoms. However, participation in this study may not directly benefit you.

6. Are there any risks?

All medical treatments involve some risk of injury or side-effects. In addition, there may be risks associated with this study that are presently unknown or unforeseeable. The risks associated with the study medication being used are well-known and are outlined below.

Study Medication: Atorvastatin

Most people who take statin drugs tolerate them very well. But some people have side-effects. The most common statin side effects include:

Common side-effects – occur in 1 to 10 of every 100 patients:

- Headache; difficulty sleeping; flushing of the skin; muscle aches, tenderness, or weakness (myalgia); drowsiness: dizziness: nausea or vomiting; abdominal cramping or pain; bloating or gas; diarrhea; constipation; rash; low levels of cells in the blood called platelets

Uncommon side-effects – occur in 1 to 10 in every 1000 patients:

- Nausea; hair loss; pins and needles sensations, such as pricking, numbness, or tingling on your skin; liver inflammation, which can cause flu-like symptoms; pancreas inflammation, which can cause stomach pain; skin problems, such as rashes or acne; sexual problems, such as erectile dysfunction or a low sex drive.

Statins also carry warnings that memory loss, mental confusion, neuropathy, high blood sugar, and type 2 diabetes are possible side-effects. It's important to remember that statins may also interact with other medications you take.

Rare and very rare side-effects – occur in less than 1 in 1000 patients:

- Myositis, which is inflammation of the muscles. The risk of muscle injury increases when certain other medications are taken with statins. For example, if there is a combination of a statin and a fibrate – another cholesterol-reducing drug – the risk of muscle damage increases greatly compared to someone who takes a statin alone.
- Elevated levels of creatine kinase (CK), a muscle enzyme that when elevated, can cause muscle pain, mild inflammation, and muscle weakness. This condition, though uncommon, can take a long time to resolve.
- Rhabdomyolysis, extreme muscle inflammation and damage. With this condition, muscles all over the body become painful and weak. The severely damaged muscles release proteins into the blood that collect in the kidneys. The kidneys can become damaged trying to eliminate a large amount of muscle breakdown caused by statin use. This can ultimately lead to kidney failure or even death. Fortunately, rhabdomyolysis is extremely rare. It happens in less than one in 10,000 people taking statins.

If you experience any unexplained joint or muscle pain, tenderness, or weakness whilst taking the study medication, you will need to contact the research office and seek early review by your doctor.

<u>Blood Collection</u>

The risks of a blood test include pain, a bruise at the point where the blood is taken, redness and swelling of the vein, infection and rarely fainting.

<u>Reproductive Risk</u>

As some studies link the use of statins with birth defects, participants in the study are advised against pregnancy. It is important that women participating in this study are not lactating mothers or pregnant, and do not become pregnant during their participation in the study. If you are a woman of child-bearing potential and there is a possibility that you are pregnant, you will be required to have a pregnancy test. You will also be required to use an effective method of contraception whilst you are participating in the study. This will be discussed with you by your doctor at the start of the study. If, at any time while you are participating in the study, you think you may have become pregnant, it is important to let your doctor know immediately.

7. Compensation for injuries or complications

If you suffer any injuries or complications as a result of this study, you should contact your doctor as soon as possible, who will assist you in arranging appropriate medical treatment. If you are eligible for public health care or medical insurance, you can receive any medical treatment required to treat the injury or complication, free of charge, as a public patient in any public hospital.

In addition, you may have the right to take legal action to obtain compensation for any injuries or complications resulting from the study. Compensation may be available if your injury or complication is sufficiently serious and is caused by unsafe drugs or equipment, or by negligence of one of the parties involved in the study (for

example, the researcher, or the treating doctor). You do not give up any legal rights to compensation by participating in this study.

8. Will taking part in this study cost me anything, and will I be paid?

In person clinic visits: You will receive $100 AUD at the baseline visit and $100AUD at the end-of-study visit (visit 5) to reimbursed for your time. You may also be reimbursed for any reasonable travel expenses related to your in-person clinic visits.

Remote participation: If you are participating remotely (via telehealth from your home), you will receive $50 AUD at the baseline visit and $50 at the end-of-study visit (visit 5) to reimbursed for your time.

9. What will happen to my test samples?

The collection of blood during this research study is a mandatory component. The tests are used to determine whether the medications have caused any unwanted side-effects. Your GP will receive any results that may be pertinent to your ongoing health. However, none of the samples will be stored or used for future research. All samples will be sent to a local laboratory for analysis and then discarded.

10. Can I have other treatments during this research project?

You may continue to take your usual treatment while participating in this study. However, you should not start the study if you have any indication or contraindication to the use of a statin for your health. Should you need treatment with a statin during the course of the study, you will be required to stop the study medication, but continue in the study until the final visit, as planned. If you routinely take over the counter medications, please discuss these with the study team.

11. Could this research study be stopped unexpectedly?

The study may be stopped unexpectedly for a variety of reasons, for example:

- unacceptable side-effects or a decision made by local regulatory health authorities.

12. What if I wish to withdraw from this research project?

If you decide to withdraw from the study, please notify a member of the research team right away and inform them of any medical problems you experienced or medications you have taken since the last study contact. This will allow the research team to further discuss any health risks or special requirements linked to withdrawing.

If you withdraw consent for further treatment, data will continue to be collected unless you specify otherwise. If you decide to leave the study, the researchers would like to keep the health information about you that has been collected. This is to help them make sure that the results of the research can be measured properly. If you decide to withdraw and do not wish for your health information that has already been collected to be used, please notify the hospital study staff.

13. What happens when the research project ends?

The medications provided during this study will not continue to be available at study completion. You will be referred back to your specialist/physician for the management of long-COVID neurological symptoms. However, statins such as atorvastatin are low cost and widely available. Your doctor or specialist can decide as to whether it is suitable for you to continue taking atorvastatin or another statin long-term. Your GP will be provided with your blood test results and interpreted neuropsychological test results.

Sometimes studies are extended so that researchers can find out more about long-term health outcomes. It is possible that the follow-up period for this study will be extended. By signing the consent form, you agree to be contacted by the researchers in the future to be invited to participate in an extension phase of the study.

14. Confidentiality / Privacy

Any identifiable information that is collected about you in connection with this study will remain confidential and will only be disclosed with your permission, or except as required by law. Only the study researchers, monitors, representatives of regulatory authorities and ethics committee, may have direct access to it. Access is required to check the accuracy of the information collected and to ensure that this trial is being carried out according to local requirements and/or regulatory guidelines.

Clinically significant findings that are relevant to your care may be shared with your GP with your permission. If the matter is urgent, we may need to contact your GP in the first instance to avoid undue delay.

The data will be analysed by qualified statisticians and academics at The George Institute, or at similar academic research institutes or at universities that The George Institute for Global Health collaborates with in Australia and other countries.

Study monitors, auditors, representatives of regulatory authorities and ethics committees, may also be granted direct access to your original medical records for verification of trial procedures and/or data.

All information transferred electronically will be stored either on the primary database (IBM Clinical Development) and backed up on to the IBM server held in the USA, or it will be stored on a research database called REDCap. REDCap is a secure, web-based database application, hosted and backed up to The George Institute for Global Health servers on a daily basis. The videoconferences will be conducted using the Zoom platform. No recordings of the videoconferences will be made. All information will be coded to protect your confidentiality and all computer records will be password protected. Trial documentation will be kept and securely archived for 15 years.

In accordance with relevant Australian and Victorian privacy and other relevant laws, you have the right to request access to your information collected and stored by the research team. You also have the right to request that any information with which you disagree be corrected. Please contact the study team member named at the end of this document if you would like to access your information.

15. Contributing to a databank

Human Research Ethics Committee (HREC) approval will be sought prior to any future use of the data. Although research studies are established with a primary purpose, it is often helpful for scientists to share the information they get from studies in order to learn more about how health is affected, and treatment works more or less in particular types of patients from different parts of the world. Combining information from different studies in one place helps them learn even more about the health and wellbeing of people, and how best to use new treatments. The collection of information is sometimes called a databank. We wish to store the coded data from this study into one or more of such databanks, where together with data from other studies, can be used to extend knowledge. This work may or may not be directly undertaken by research personnel associated with this study but in most cases is undertaken by staff who work for research institutes or universities. Information will be contributed to such databanks in a way that you cannot be identified. The location of such databanks will be at The George Institute, or at similar academic research institutes or at universities that The George Institute for Global Health collaborates with in Australia and other countries. It is not possible to determine how long the study data will be stored in any databanks.

The consent form for this study includes an option for you to decide whether or not you wish to consent only to your information being used for the purposes of this study, or if you also consent to your coded information being used for extended related research and therefore consent to your information being stored in a databank.

16. What happens with the results?

All information collected from you for this study will be stored electronically in a database maintained by The George Institute for Global Health of the University of New South Wales in Australia. It is intended for the results of this study to be presented or published at medical conferences and in scientific journals.

In any publication, information will be provided in such a way that you cannot be identified. Results of the study will be provided to you, if you wish. By signing the consent form, you agree to your data being included in the results published for this study.

Further information

When you have read this information, the Principal Investigator and/or Research Coordinator will discuss it with you further and answer any questions you may have. If you would like to know more at any stage, please feel free to contact:

Site Principal Investigator: ______________________________

[Insert site-specific details]

Complaints: ______________________________

[Insert site-specific procedures]

Ethics Approval

All research involving humans is reviewed by an independent group of people called a Human Research Ethics Committee (HREC) or Institutional Review Board (IRB). This study has been approved by the Ethics Review Committee (RPAH Zone) of the Sydney Local Health District. Any person with concerns or complaints about the conduct of this study should contact the Executive Officer on 02 9515 6766 and quote protocol number X21-0113.

The conduct of this study at the [name of study site] has been authorised by the [name of Local Health District/Governance Office]. Any person with concerns or complaints about the conduct of this study may also contact the Research Governance Officer [or other officer] on [telephone number] and quote protocol number [insert local protocol number].

This study will be carried out in accordance with the *National Statement on Ethical Conduct in Human Research (2007, updated May 2018)*. This statement has been developed to protect the interests of people who agree to participate in human research studies.

Thank you for taking the time to consider this study. If you wish to take part in it, please sign the attached consent form. This information sheet is for you to keep.

Consent to Participate in Research

**Title**	Statin TReatment for COVID-19 to Optimise NeuroloGical recovERy
**Short Title**	STRONGER
**Protocol Number**	N/A
**Study Sponsor**	The George Institute for Global Health
**Principal Investigator**	______________________________ *[insert name]*

I, ____

[name]

of ____

[address]

have read and understood the Information for Participants on the above-named research study.

1. I have been made aware by ________________________________ ("the researcher") of the procedures involved in the study, time involved, including any known or expected inconvenience, risks, discomfort or potential side-effects and of their implications as far as they are currently known.

2. I understand that the researcher will conduct this study in a manner conforming to ethical and scientific principles set out by the National Health and Medical Research Council (NHMRC) of Australia and the Good Clinical Research Practice Guidelines of the Therapeutic Goods Administration.

3. I acknowledge that I have been given time to consider the information and to seek other advice.

4. I acknowledge that refusal to take part in this study will not affect the usual treatment of my condition.

5. I acknowledge that I am volunteering to take part in this study, and I may withdraw at any time.

6. I understand that my participation in this study will allow the researchers and others, as described in the Information for Participants, to have access to my medical record, and I agree to this.

7. I consent to my contact details being provided to the courier service to facilitate delivery of the study drugs.

8. I understand that any blood samples collected will only be used for this research project, as described in the relevant section of the Participant Information Sheet.

9. I acknowledge that this research has been approved by the Sydney Local Health District Human Research Ethics Committee.

10. I acknowledge that any regulatory authorities may have access to my medical records concerning my disease and treatment for the purposes of this project. However, I understand my identity will not be disclosed to anyone else or in publications or presentations.

11. I understand that I may be contacted after the end of this study to be invited to participate further for assessment of my health and wellbeing in the longer term.

12. I would like to receive a copy of the study results when they become available. My email address is: ________________

13. I understand that I will be given a signed copy of this document and the Participant Information sheet to keep.

Circle *YES* or *NO* in response to the following statement

14. I agree for the information being collected about me during this study to also be contributed to one or more databanks for the purpose of extended research and I understand that any such data will be stored in such a way that will not identify me.

YES ***NO***

**Name of Participant**	______________________________________ *(please print – First name / Family name)*		
**Signature**	______________________	**Date**	______________

<u>Declaration by Witness</u>

I have witnessed and certify the Participant's verbal consent for he/she to voluntarily agree to participate in this research study.

**Signature of Impartial Witness**	______________________	**Date**	______________
(to be completed only if the participant cannot sign the patient information sheet)			
**Printed name of Impartial Witness**	______________________	**Relationship to the Participant**	______________
The Participant's confirmation is attested by the above signature of an Impartial Witness			

<u>Declaration by Study Doctor/Senior Researcher[†]</u>

I have given a verbal explanation of the research project; its procedures and risks and I believe that the participant has understood that explanation.

**Name of Study Doctor/ Senior Researcher[†]**	______________________________________ *(please print – First name / Family name)*

Signature ______________________________ **Date** ____________________

†A senior member of research team must provide the explanation of, and information concerning, the research project.

Note: All parties signing the consent section must date their own signature.

Form for Withdrawal of Participation

**Title**	Statin TReatment for COVID-19 to Optimise NeuroloGical recovERy (STRONGER)
**Short Title**	STRONGER
**Project Sponsor**	The George Institute for Global Health, Sydney, Australia
**Coordinating Principal Investigator**	Professor Craig Anderson

Declaration by Participant

I wish to withdraw from participation in the above research project and understand that such withdrawal will not affect my routine treatment or my relationship with those treating me.

Name of Participant (please print) ______________________________

Signature ______________________ Date ______________________

Consent provided to use data collected up to the date of withdrawal: Yes ☐ No ☐

In the event that the participant's decision to withdraw is communicated verbally, the Study Doctor/Delegate/Study Coordinator will need to provide a description of the circumstances below:

Declaration by Study Doctor/Study Coordinator †

I have given a verbal explanation of the implications of withdrawal from the research project and I believe that the participant has understood that explanation.

Name of Study Doctor/
Study Coordinator (please print) ______________________________

Signature ______________________ Date ______________________

Note: All parties signing the withdrawal section must date their own signature.

Master Participant Information Sheet/Consent Form

Title	Statin TReatment for COVID-19 to Optimise NeuroloGical recovery (STRONGER) Biomarker & MRI Sub-Study
Short Title	STRONGER Sub-Study
Protocol Number	N/A
Study Sponsor	The George Institute for Global Health (TGI), University of New South Wales, Sydney, Australia
Coordinating Principal Investigator / Principal Investigator	______________________________ *[insert name]*

1. Introduction

You have consented to participate in a research study called STRONGER. This study aims to determine the effect of using a statin (a cholesterol-lowering medication) for improving the effects of "long COVID" symptoms on the brain. The treatment in this study involves the use of a commonly used statin called atorvastatin. As part of the STRONGER study, we also invite people to participate in the STRONGER Biomarker & MRI Sub-Study which aims to further assess the effectiveness of the study treatment on decreasing any signs of inflammation in the brain and blood. Magnetic Resonance Imaging (MRI) will be used to look at inflammation markers in the brain and additional blood samples will be taken to look at so-called neurodegenerative biomarkers.

The STRONGER MRI and Biomarker Sub-Study is being coordinated jointly by researchers from The George Institute for Global Health (TGI), University of New South Wales, the Departments of Electrical and Computer Systems Engineering, and Neuroscience (Alfred Hospital Precinct) and Monash University, Melbourne, Australia.

2. Background and purpose of the STRONGER Biomarker & MRI Sub-Study

There is increasing evidence to indicate that low levels of inflammation in the body can affect the strength and function of blood vessels, including those in the brain. New technology that uses MRI can detect early signs of inflammation in the brain, and this causes the release of proteins from the brain into the blood. These types of abnormalities have been shown to occur with other injuries to brain, including in people with, or at risk of, Alzheimer's Disease.

3. Why have I been chosen?

You have been asked to participate in the STRONGER Biomarker & MRI Sub-Study because you are currently participating in the STRONGER study. This sub-study will be carried out in selected medical centres in Australia and Chile, where a specific type of MRI can be conducted. A total of approximately 220 people will be invited to participate.

4. What will my participation in the STRONGER Biomarker & MRI Sub-Study involve?

Participation involves undergoing 2 MRI brain scans. These will be done at the following times:

1. Visit 2 – either the same day or within the next 7-10 days of the screening/baseline clinical assessment (depending on booking availability)
2. Visit 5 – the End of Study visit, 12 months (± 1 month) from starting the study

You will be asked to provide a 28.5ml blood sample (note – the total amount of blood needed for the main study and this sub-study is 48.5ml) for laboratory measurement. Bloods should be drawn in the morning.

Each scan will take approximately 45mins to complete. The appointments for your MRI scans will be made for you by the STRONGER study coordinator/staff member at a time that suits you. The MRI scans will be conducted at a designated MRI centre at or near the study site that you attend your STRONGER study visits.

If you agree to participate in this study, you will be asked to sign the Participant Consent Form to confirm that you understand the purpose and what is involved in the STRONGER Biomarker & MRI Sub-Study and that you are free to withdraw at any time.

You will be informed in a timely manner if information becomes available that may be relevant to your willingness to continue participation in the STRONGER Biomarker & MRI Sub-Study.

5. What are the alternatives to participation?

Participation in this research study is voluntary. You can discuss the study with your general practitioner or medical specialist before you decide whether or not to take part.

6. Are there any benefits?

The Sub-Study aims to generate further medical knowledge and to improve the management of patients with long COVID-19. However, participation in the STRONGER Biomarker & MRI Sub-Study may not directly benefit you.

7. Are there any risks?

All medical treatments involve some risk of injury or side-effects. In addition, there may be risks associated with the STRONGER Biomarker & MRI Sub-Study that are presently unknown or unforeseeable. The risks associated with MRI scans are well known and are outlined below.

MRI scanning can sometimes cause mild anxiety. However, this usually lessens when testing begins, and the researchers are trained to deal with these situations. If you are claustrophobic, that is you suffer extreme fear of enclosed spaces, it may not be appropriate for you to undergo MRI scanning. Sometimes a mild sedative can be used to relax you. This might mean that we have to re-schedule your visit for the MRI if you decide to proceed with this investigation.

MRI scans involves a strong, static magnetic field that can cause the following safety concerns:

- the field will attract magnetic objects (from small items such as keys and cell phones, a pacemaker or artificial hip joint, to large, heavy items such as oxygen tanks) which may cause these objects to become projectiles. By carefully screening people and removing any metal objects before entering the MRI environment from the magnetic field will minimise this risk. You will not have the MRI if you have a metallic implant.
- during the MRI, loud knocking noises are heard. These noises may harm hearing if adequate ear protection is not used. Hearing protection is provided to minimise this risk.
- the radiofrequency energy used during the MRI scan could lead to minor heating of the body. The potential for heating is greater during long MRI examinations and is less likely during a 45-minute scan planned for this Sub-Study.

<u>Blood Collection</u>

The risks of a blood test include pain, a bruise at the point where the blood is taken, redness and swelling of the vein, infection, and rarely fainting.

8. What happens if the MRI scan reveals an abnormality that I was not aware of?

The procedures used in this study are not diagnostic. However, MRI scans may incidentally reveal an unexpected abnormality. All MRI scans are reviewed by a clinical radiologist, and any abnormality is reported. The clinical radiologist will provide a report on the abnormality, including a recommendation as to whether clinical follow-up is necessary.

After your scan, the images will be sent to research specialists located at Monash University in Melbourne, Australia who will examine them for measurement and detection of any abnormalities. These tests of inflammation in the brain are not done routinely during a clinical examination and are not widely available for patients as part of normal practice. This will not be done on the day of your scan. You should be aware that because the images are taken for a specific research purpose, not all abnormalities that might be detected by other MRI scans are necessarily seen.

In the unlikely event that an abnormality for which treatment or follow-up is recommended, or other information relevant to your health is found on the brain MRI, we will send a report to your regular doctor. We will also contact you with further advice about the next steps.

9. Compensation for injuries or complications

If you suffer any injuries or complications as a result of the STRONGER Biomarker & MRI Sub-Study, you should contact your regular doctor as soon as possible, who will assist you in arranging appropriate medical treatment. If you are eligible for public health care or medical insurance, you can receive any medical treatment required to treat the injury or complication, free of charge, as a public patient in any public hospital.

In addition, you may have the right to take legal action to obtain compensation for any injuries or complications resulting from the STRONGER Biomarker & MRI Sub-Study. Compensation may be available if your injury or complication is sufficiently serious and is caused by unsafe drugs or equipment, or by negligence of one of the parties involved in the STRONGER Biomarker & MRI Sub-Study (for example, the researcher or the treating doctor). You do not give up any legal rights to compensation by participating in STRONGER Biomarker & MRI Sub-Study. If you are eligible for medical treatment, you can receive this for your injury or complication free of charge as a public patient in any public hospital.

10. Will taking part cost me anything?

Participation in the Biomarker & MRI Sub-study will not cost you anything. However, you will be reimbursed for reasonable travel expenses to attend the scanning centre if this is at a different location to the study site.

11. What will happen to my blood samples and MRI scans?

Bloods will be collected at the clinic that you attend, the samples will be processed and stored at this site until the end of the study. It will then be sent to Departments of Electrical and Computer Systems Engineering, and Neuroscience (Alfred Hospital Precinct) and Monash University, Melbourne, Australia, where your blood will be analysed for the inflammatory biomarkers. These samples will also be stored for future exploratory analyses, and only for the purposes of examining inflammation and degeneration in the brain.

Your codified MRI scans will be recorded on a computer and the de-identified data will be saved on a server at the Monash University Melbourne, Australia. Your study site will also store a hard copy of your scan on a CD-ROM. All scans will be stored for at least 15 years following the completion of the STRONGER Biomarker & MRI Sub-Study.

12. Can I have other treatments while participating in the STRONGER Biomarker & MRI Sub-Study?

You may continue with usual treatments throughout the Biomarker & MRI Sub-Study. However, should you require implantation of any metallic devices, you must notify your STRONGER study doctor and you must withdraw from further participation in the STRONGER Biomarker & MRI Sub-Study.

13. What if I wish to withdraw from the research project?

Participation in this sub-study is voluntary. If you do not wish to take part, you do not have to. If you decide to take part and later change your mind, you are free to withdraw from the project at any stage.

If you decide to withdraw from the sub-study, please notify a member of the research team right away and inform them of any medical problems you experienced or medications you have taken since the last study contact. This will allow the research team to further discuss any health risks or special requirements linked to withdrawing.

14. What happens when the STRONGER Biomarker & MRI Sub-Study ends?

When the STRONGER Biomarker & MRI Sub-Study is completed, copies of the scans may be made available to you or your treating physician/GP on request.

15. Confidentiality / Privacy

Any identifiable information that is collected about you in connection with the STRONGER Biomarker & MRI Sub-Study will remain confidential and will be disclosed only with your permission, or except as required by law. Only the STRONGER study and the STRONGER Sub-Study researchers, monitors, representatives of relevant regulatory authorities and ethics committees, may have direct access to it. Access is required to check the accuracy of the information collected and to ensure that this trial is being carried out according to local requirements and/or regulatory guidelines.

The data will be analysed by qualified statisticians and academics at The George Institute, or at similar academic research institutes or at universities that The George Institute for Global Health collaborates with in Australia and other countries.

The STRONGER study monitors, auditors, representatives of regulatory authorities and ethics committees may also be granted direct access to your original medical records for verification of trial procedures and/or data.

All information transferred electronically will be stored either on the primary database (IBM Clinical Development) and backed up on to the IBM server held in the USA, or it will be stored on a research database called REDCap. REDCap is a secure, web-based database application, hosted and backed up to The George

Institute for Global Health servers on a daily basis. All information will be coded to protect your confidentiality and all computer records will be password protected. Trial documentation will be kept and securely archived for 15 years.

Some of the information in the STRONGER Biomarker & MRI Sub-Study may be collected by trained researchers appointed by The George Institute who will work independently from those managing the STRONGER Biomarker & MRI Sub-Study. By signing this consent form, you are agreeing for your contact details to be stored in a secure password-protected database that is accessible only by the staff at the hospital and by the designated independent researchers who may contact you to find out whether you have had any serious illness or whether you have been admitted to hospital, during the study. This database will be separate from the database containing your STRONGER Biomarker & MRI Sub-Study data, and these will not be linked in any way so that confidentiality and de-identification of the information collected about you for the purpose of STRONGER Biomarker & MRI Sub-Study will be maintained.

16. What happens with the results?

All information collected from you for the STRONGER Biomarker & MRI Sub-Study will be stored electronically in databases maintained by The George Institute and the Departments of Electrical and Computer Systems Engineering, and Neuroscience (Alfred Hospital Precinct) and Monash University, Melbourne, Australia. It is intended for the results of the STRONGER Biomarker & MRI Sub-Study to be presented or published at medical conferences and in scientific journals. In any publication, information will be provided in such a way that you cannot be identified. Results of the STRONGER Biomarker & MRI Sub-Study will be provided to you, if you wish. By signing the consent form, you agree to your data being included in the results published for the STRONGER Biomarker & MRI Sub-Study.

17. Further Information

When you have read this information, the Principal Investigator and/or Research Coordinator will discuss it with you further and answer any questions you may have. If you would like to know more at any stage, please feel free to contact:

Site Principal Investigator: ____

[Insert site specific details]

Complaints: ____

[Insert site specific procedures]

Ethics Approval

All research involving humans is reviewed by an independent group of people called a Human Research Ethics Committee (HREC) or Institutional Review Board (IRB). The STRONGER Biomarker & MRI Sub-Study has been approved by the Ethics Review Committee (RPAH Zone) of the Sydney Local Health District. Any person with concerns or complaints about the conduct of this study should contact the Executive Officer on 02 9515 6766 and quote protocol number X21-0113.

The conduct of the STRONGER Biomarker & MRI Sub-Study at the [name of study site] has been authorised by the [name of Local Health District/Governance Office]. Any person with concerns or complaints about the conduct of the Sub-Study may also contact the Research Governance Officer [or other officer] on [telephone number] and quote protocol number [insert local protocol number].

The STRONGER Biomarker & MRI Sub-Study will be carried out in accordance with the *National Statement on Ethical Conduct in Human Research (2007, updated March 2014)*. This statement has been developed to protect the interests of people who agree to participate in human research studies.

Thank you for taking the time to consider the STRONGER Biomarker & MRI Sub-Study. If you wish to take part in it, please sign the attached consent form. This information sheet is for you to keep.

Consent to Participate In Research

**Title**	Statin TReatment for COVID-19 to Optimise NeuroloGical recovery (STRONGER) Biomarker & MRI Sub-Study
**Short Title**	STRONGER
**Protocol Number**	N/A
**Project Sponsor**	The George Institute for Global Health (TGI), University of New South Wales, Sydney, Australia
**Coordinating Principal Investigator / Principal Investigator**	______________________________ *[insert name]*

I, ____
[name]

of ____
[address]

have read and understood the Information for Participants on the above-named STRONGER Biomarker & MRI Sub-Study.

1. I have been made aware by _________________________ ("the researcher") of the procedures involved in the STRONGER Biomarker & MRI Sub-Study, time involved, including any known or expected inconvenience, risks, discomfort or potential side-effects and of their implications as far as they are currently known.

2. I understand that the researcher will conduct the STRONGER Biomarker & MRI Sub-Study in a manner conforming to ethical and scientific principles set out by the National Health and Medical Research Council (NHMRC) of Australia and the Good Clinical Research Practice Guidelines of the Therapeutic Goods Administration.

3. I acknowledge that I have been given time to consider the information and to seek other advice.

4. I acknowledge that refusal to take part in the STRONGER Biomarker & MRI Sub-Study will not affect the usual treatment of my condition or continuing participation in the STRONGER Study.

5. I acknowledge that I am volunteering to take part in the STRONGER Biomarker & MRI Sub-Study, and I may withdraw at any time.

6. I understand that any MRI scans and blood samples collected will only be used for the STRONGER Biomarker & MRI Sub-Study, as described in the relevant section of the Participant Information Sheet.

7. I acknowledge that the STRONGER Biomarker & MRI Sub-Study has been approved by: the Sydney Local Health District Human Research Ethics Committee.

8. I acknowledge that any regulatory authorities may have access to my medical records concerning my disease and treatment for the purposes of the STRONGER Biomarker & MRI Sub-Study. However, I understand my identity will not be disclosed to anyone else or in publications or presentations.

9. I understand that I will be given a signed copy of this document and the Participant Information Sheet to keep.

Name of Participant ____

(please print – First name / Family name)

Signature ______________________ **Date** ______________

Declaration by Witness

I have witnessed and certify the Participant's verbal consent for him/her to voluntarily agree to participate in the STRONGER Biomarker & MRI Sub-Study.

Signature of Impartial Witness ______________________ **Date** ______________

(to be completed only if the participant cannot sign the patient information sheet)

Printed name of Impartial Witness ______________________ **Relationship to the Participant** ______________

The Participant's confirmation is attested by the above signature of an Impartial Witness

Declaration by Study Doctor/Senior Researcher†

I have given a verbal explanation of the STRONGER Biomarker & MRI Sub-Study; its procedures and risks and I believe that the participant has understood that explanation.

Name of Study Doctor/ Senior Researcher† ____

(please print – First name / Family name)

Signature ______________________ **Date** ______________

†A senior member of research team must provide the explanation of, and information concerning, the research project.

Note: All parties signing the consent section must date their own signature.

Form for Withdrawal of Participation

**Title**	Statin TReatment for COVID-19 to Optimise NeuroloGical recovERy (STRONGER)
**Short Title**	STRONGER
**Project Sponsor**	The George Institute for Global Health, Sydney, Australia
**Coordinating Principal Investigator**	Professor Craig Anderson

Declaration by Participant

I wish to withdraw from participation in the sub-study of the above research project and understand that such withdrawal will not affect my routine treatment or my relationship with those treating me.

Name of Participant (please print) ______________________

Signature ______________________ Date ______________________

Consent provided to use data collected up to the date of withdrawal: Yes ☐ No ☐

In the event that the participant's decision to withdraw from the sub-study is communicated verbally, the Study Doctor/Delegate/Study Coordinator will need to provide a description of the circumstances below:

Declaration by Study Doctor/Study Coordinator †

I have given a verbal explanation of the implications of withdrawal from the sub-study and I believe that the participant has understood that explanation.

Name of Study Doctor/
Study Coordinator (please print) ______________________

Signature ______________________ Date ______________________

Note: All parties signing the withdrawal section must date their own signature.

.

Documento informativo y formulario de consentimiento para el participante

**Título**	Tratamiento con estatina para el COVID-19, con el fin de optimizar la recuperación neurológica
**Título breve**	STRONGER
**Número de protocolo**	N/A
**Patrocinador del estudio**	George Institute for Global Health (TGI), Universidad New South Wales, Sídney, Australia
**Investigador responsable en CAS UDD**	Dra. Paula Muñoz

1. Introducción

Usted está siendo invitado a participar en un estudio de investigación llamado STRONGER. Este estudio apunta a determinar el efecto de utilizar una estatina (un medicamento para reducir el nivel de colesterol) para mejorar los efectos de síntomas "a largo plazo del COVID" en el cerebro. El tratamiento en este estudio involucra el uso de una estatina utilizada comúnmente, llamada atorvastatina. La razón por la que se le ha invitado a participar en este estudio es porque anteriormente tuvo COVID-19 y ha tenido algunos síntomas neurológicos prolongados, como problemas de memoria, concentración, estado de ánimo, sueño y/o fatiga.

Un grupo de investigadores internacionales realizará el estudio, que involucrará a 410 participantes en Australia, principalmente de los estados de Victoria y Nueva Gales del Sur, Chile y Sudamérica, Hong Kong y, posiblemente, otros países. Este estudio ha recibido su fuente principal de financiamiento de la Comisión Nacional de Salud e Investigación Médica (NHMRC, por sus siglas en inglés) de Australia, así como del Fondo Futuro para la Investigación Médica (MRFF, por sus siglas en inglés), y está bajo la coordinación a nivel internacional del George Institute for Global Health de la Universidad de Nueva Gales del Sur, Australia.

Este documento informativo y formulario de consentimiento para el participante le informa sobre el estudio de investigación, explicando los exámenes y los tratamientos que están involucrados en él. Saber lo que implica el estudio le ayudará a decidir si es que desea participar de él o no.

Por favor, lea esta información cuidadosamente. Haga preguntas sobre cualquier cosa que no entienda o sobre la que quiera saber más. Antes de decidir si participar o no, es posible que desee hablar sobre ello con un pariente, amigo o con su médico.

La participación en esta investigación es voluntaria. Si no desea participar, no tiene que hacerlo. Recibirá la mejor atención posible, ya sea que desee participar o no. Cualquiera sea su decisión, por favor cuente con la seguridad de que esto no afectará su tratamiento médico ni su relación con el personal que le está atendiendo.

COMITÉ ÉTICO CIENTÍFICO
Facultad de Medicina
Clínica Alemana - Universidad del Desarrollo
Centro de Bioética
23.03.2023

.

A veces, durante el transcurso de un estudio, hay nueva información disponible sobre el tratamiento que se está estudiando. Mientras participe en este estudio, se le mantendrá informado sobre cualquier nuevo hallazgo significativo que pudiera afectar su voluntad de continuar en el estudio.

Si decide participar en este estudio, se le pedirá firmar la sección sobre consentimiento, incluida en este formulario. Al firmar este formulario, nos está informando lo siguiente:

- que entiende lo que ha leído;
- que consiente a participar en el proyecto de investigación;
- que consiente los exámenes y tratamientos que se describen en este documento informativo; y
- que consiente el uso de su información personal y de salud, como se describe en este documento.

Se le entregará una copia de este documento informativo y formulario de consentimiento para el participante, para que lo guarde.

2. ¿Cuál es el propósito de esta investigación?

El propósito de esta investigación es ver si es que el tratamiento con estatina (un medicamento para bajar los niveles de colesterol), además del cuidado estándar, es mejor para mejorar los síntomas neurológicos relacionados al COVID-19, si se compara con el cuidado estándar por sí solo.

El medicamento que se utilizará en esta prueba se llama atorvastatina. La atorvastatina es una estatina, un tipo de fármaco que los médicos recetan a menudo para ayudar a reducir los niveles de colesterol en la sangre. Al reducir estos niveles, las estatinas ayudan a prevenir ataques al corazón e infartos cerebrales. Sin embargo, las estatinas también trabajan por otros mecanismos para reducir la inflamación en las arterias y en otras partes del cuerpo. Debido a que la atorvastatina es capaz de penetrar en el cerebro y reducir la inflamación en el cuerpo, podría ofrecer beneficios para las personas que han sufrido de COVID-19.

Este medicamento está aprobado por diferentes organismos normativos, como la Administración de Bienes Terapéuticos (TGA, por sus siglas en inglés) en Australia y la Administración de Alimentos y Medicamentos (FDA, por sus siglas en inglés) de los Estados Unidos, así como también en Chile, para el tratamiento de personas con altos niveles de colesterol. Esencialmente, el estudio STRONGER está investigando si es que el uso de una estatina puede ayudar a mejorar la función cerebral y a reducir los efectos a largo plazo causados por el COVID-19. **La atorvastatina aún no ha sido aprobada ni en Australia ni en Chile para el tratamiento de los síntomas neurológicos causados por el COVID-19. Este estudio será aprobado por el Instituto de Salud Pública de Chile. Esto permitirá a los investigadores usar este producto con fines de investigación médica, una vez que la investigación haya sido evaluada y aprobada por un Comité de Ética de Investigación en Seres Humanos (HREC, por sus siglas en inglés).**

El *COVID-19*, una enfermedad infecciosa emergente causada por el síndrome respiratorio agudo derivado del coronavirus 2 (SARS-CoV-2), ha afectado a casi 100 millones de personas alrededor del mundo, y ha ocasionado más de 1 millón 800 mil de muertes desde el primer brote en China, a finales de 2019. Los científicos y las personas del área médica han, ahora, descrito bien los síntomas y las complicaciones de quienes recién se han infectado con COVID-19; sin embargo, hay incertidumbre sobre sus consecuencias a largo plazo en la salud de los pacientes. Hay un reconocimiento cada vez mayor de que muchos pacientes tienen síntomas persistentes, como problemas respiratorios prolongados, menos energía, fuerza muscular, memoria y estado de ánimo.

COMITÉ ÉTICO CIENTÍFICO
Facultad de Medicina
Clínica Alemana - Universidad del Desarrollo
Centro de Bioética
23.03.2023

.
Estos síntomas no se limitan a las personas que han sufrido de una enfermedad severa y que estuvieron hospitalizados por COVID-19. Los estudios han demostrado que hasta tres cuartos de los pacientes reportan al menos un síntoma varios meses después del inicio de la infección. Los síntomas más comunes de un "COVID extenso" son fatiga, debilidad muscular, problemas de sueño y ansiedad o depresión. Aproximadamente 5% a 10% de las personas reportan tener síntomas neurológicos, como "confusión mental", cefaleas, baja concentración y mareos.

3. ¿Qué involucra la participación en esta investigación?
La duración de su participación en este estudio es de 12 meses.

Los participantes potencialmente elegibles que expresen interés en el estudio serán contactados por el coordinador del estudio para discutir los requisitos del mismo, evaluar brevemente la elegibilidad y compartir una copia del consentimiento para que lo revise.

Para confirmar su elegibilidad, los participantes avanzarán a través de un proceso de selección de 2 etapas que incluye:

Visita 1: teleconsulta (telefónica o por videollamada) y la realización de una evaluación preliminar de consentimiento en línea, y;

Visita 2: evaluación clínica y cognitiva más detallada en persona para confirmar la elegibilidad (esta visita se puede realizar por videoconferencia si es necesario).

Visita 1 : Screening preliminar - teleconsulta

Se le invitará a asistir a una teleconsulta (por teléfono o videoconferencia) para completar el proceso de consentimiento informado y, después de otorgar este consentimiento, completar los procedimientos preliminares de selección.

En esta visita de evaluación preliminar, un miembro capacitado del equipo del estudio, generalmente un médico, enfermero o coordinador de investigación, le hará una serie de preguntas sobre su historial médico y sus medicamentos actuales. También completarán un cuestionario con usted y evaluarán su memoria y pensamiento, lo que se llama "cognición" (esto significa el proceso de comprender cómo recuerda las cosas, cómo resolver problemas, etc.).

La información recolectada sobre usted en esta Visita 1 determinará si es posible que usted pueda seguir en el estudio. Si lo es, se le pedirá que asista a una visita de selección adicional.

Completar el proceso de selección preliminar y firmar el formulario de consentimiento no garantiza su enrolamiento en el estudio. Más bien, permite que el equipo de estudio inicie discusiones e investigaciones para evaluar su idoneidad para participar.

Esta visita durará aproximadamente 30-45 minutos.

Visita 2 : Visitas de investigación de antecedentes/aleatorización (visita clínica) En la visita 2 se le invitará a asistir a una visita presencial durante la mañana. Se le solicitará asistir en ayunas (sin haber comido nada por 8 horas), para completar el proceso de investigación de antecedentes y, si resulta ser elegible, para completar

COMITÉ ÉTICO CIENTÍFICO
Facultad de Medicina
Clínica Alemana - Universidad del Desarrollo
Centro de Bioética
23.03.2023

.

la evaluación. Se le pedirá una muestra de sangre de 20 ml, para realizar mediciones de laboratorio, que se recogerán en la clínica o en un laboratorio local. Los análisis de sangre requeridos para esta visita también se pueden recolectar antes de esta visita en un laboratorio designado por el estudio para permitir que los resultados de los análisis de sangre estén disponibles para esta visita y / o para permitir flexibilidad si las visitas continuas se realizan de forma remota.

En esta visita de revisión de antecedentes, un miembro entrenado del equipo de estudio, usualmente un médico, un(a) enfermero(a) o el coordinador de la investigación, le realizará una serie de preguntas acerca de su historial médico y de los medicamentos que toma actualmente. También pasará por un breve examen físico, para recolectar evaluaciones clínicas que incluyen la medición de su presión arterial y su ritmo cardíaco, su peso y estatura. También, una neuropsicóloga de nuestro equipo realizará una evaluación de su memoria y pensamiento, que se llama "cognición" (este es el proceso para entender cómo recuerda las cosas, cómo resuelve problemas, etc.), evaluará su estado de ánimo, su bienestar en general y se incluirán cuestionarios para evaluar su calidad de vida y nivel de actividad física en relación con su salud y los servicios de salud que ha utilizado recientemente mediante una serie de cuestionarios.

Al final de su primera visita, su médico o enfermero(a) del estudio confirmará si usted es elegible para seguir participando en él. Si lo es, y si desea continuar siendo parte de él, entonces usted será "aleatorizado", como en un juego de cara o sello, para recibir el tratamiento del estudio. Tendrá un 50% de probabilidad de recibir:

- cuidado estándar (es decir, no recibirá medicamento por el estudio y puede seguir con el indicado por su médico de cabecera si aplica); o
- cuidado estándar + 40 mg de atorvastatina

Ambas ramas del estudio pasarán por las mismas evaluaciones en cada visita.

A veces, no sabemos cuál tratamiento es el mejor para tratar una condición médica. Para averiguarlo, tenemos que comparar tratamientos diferentes. Ponemos a las personas en grupos y le damos a cada grupo un tratamiento diferente. Es decir, un grupo recibirá el medicamento y otro no. Luego, los resultados se comparan para ver si uno es mejor que el otro. Para intentar asegurarse de que los grupos sean iguales, cada participante se pone en un grupo de manera aleatoria.

Si usted queda en la rama del estudio con el medicamento, se le pedirá que retire la medicación en el centro del estudio. En caso de que la visita no pueda ser realizada en forma presencial, el medicamento será enviado a domicilio a través de la droguería o farmacia. Un miembro del equipo del estudio le enviará sus medicamentos por correo certificado, o usted lo puede ir a buscar al lugar donde se realizará la investigación, si es más adecuado. El equipo de investigación asignará una "droguería o farmacia" que será la que proveerá el medicamento del estudio, a la que se le entregarán sus datos de contacto, para facilitar la entrega de dichos medicamentos, en el caso de que usted entregue su consentimiento. Usted tendrá que entregar las especificaciones del despacho (es decir, que el agente de correo le entregue los medicamentos en su hogar o que se le vuelva a despachar si usted no se encuentra). Se le dará la instrucción de tomar una tableta del medicamento del estudio, una vez al día, que puede ser ingerida junto o no a una comida. Puede tomarse la tableta junto a sus otros medicamentos a la misma hora, todos los días. Recibirá instrucciones específicas sobre la toma del medicamento del estudio. Si no toma una dosis y está dentro de 12 horas de la hora en la que

COMITÉ ÉTICO CIENTÍFICO
Facultad de Medicina
Clínica Alemana - Universidad del Desarrollo
Centro de Bioética
23.03.2023

.
usualmente toma el medicamento, ingiérala inmediatamente. Si han pasado más de 12 horas, tome el medicamento el día siguiente, a la hora usual. Se le pedirá que traiga los medicamentos del estudio que le sobren en cada visita clínica (en las visitas a los 6 y 12 meses), para que el personal del estudio lo revise. En caso de cualquier requerimiento de devolución de medicamentos en cualquier momento del estudio, tendrá que ponerse en contacto con nosotros para coordinar la devolución de éste.

Después de esta visita, se le entregará un reloj de actigrafía que deberá usar durante un período de 7 días en las próximas 2 semanas después de esta visita (después de la asignación al azar). Este periodo de 7 días debiera incluir 2 días del fin de semana y 5 días de la semana, ej. de lunes a domingo. Este reloj registrará su actividad física y su sueño, y será necesario que lo devuelva a una oficina central para descargar los datos.

Si usted queda en la rama del estudio sin medicamento, además de las evaluaciones del estudio usted continuará sus controles habituales y el tratamiento que indique su médico de cabecera.

Sub estudio de biomarcadores y de imagen por resonancia magnética

Se le podría invitar a participar en un sub estudio para la recolección y almacenamiento de muestras de sangre para estudios futuros de biomarcadores neurodegenerativos, y un escáner cerebral que usa magnetos, llamado "imágenes por resonancia magnética (IRM)", que tomará alrededor de 45 minutos en hacerse. Se le pedirá que firme un consentimiento informado independiente si decide participar de este sub estudio.

En total, esta visita tomará aproximadamente 2 a 3 horas.

Visita 3 : seguimiento a las 6 semanas (visita telefónica)

Durante esta visita telefónica, se le pedirá responder algunas preguntas sobre su salud y sus medicamentos. Se le harán preguntas sobre su adherencia al medicamento del estudio, si es que está en la rama del medicamento del estudio, y que describa cualquier efecto secundario que haya tenido.

Esta visita durará aproximadamente entre 15 y 30 minutos.

Visita 4 : visita de seguimiento a los seis meses (visita clínica)

Se le harán preguntas sobre cómo ha tolerado el medicamento del estudio, si es que está en la rama del medicamento del estudio, y sobre cualquier cambio en sus otros medicamentos. Se medirá su presión arterial y su ritmo cardíaco. . Se le pedirá que complete algunos cuestionarios relacionados con su nivel actual de actividad física y estilos de vida. Se le pedirá que complete algunos test cognitivos, algunos de estos relacionados a su memoria y estado de ánimo. Se le realizarán algunas preguntas relación a su uso de servicios médicos. Se verificarán los medicamentos del estudio que usted devolvió. Se le harán preguntas sobre su adherencia al medicamento del estudio y se le pedirá que describa cualquier efecto secundario que pueda haber experimentado. Después de esta visita, se le entregará la siguiente partida del medicamento del estudio.

Esta visita tomará aproximadamente 2 a 3 horas.

Visita 5 : seguimiento a los 12 meses (visita clínica)

COMITÉ ÉTICO CIENTÍFICO
Facultad de Medicina
Clínica Alemana - Universidad del Desarrollo
Centro de Bioética
23.03.2023

.

La visita de seguimiento final se realizará en el centro clínico e involucrará un conjunto de evaluaciones. Se le realizarán exámenes clínicos (presión arterial, ritmo cardíaco y peso) y se le pedirá responder algunas preguntas sobre su salud y sus medicamentos, así como algunos cuestionarios completos relacionados con su salud que incluye el nivel de actividad física y pruebas cognitivas (memoria y estado de ánimo). Si se encuentra en la rama del medicamento del estudio, se verificará el medicamento del estudio que devolvió y se le harán preguntas sobre su adherencia a este, además de pedirle que describa cualquier efecto secundario que pudiera haber experimentado. Se le pedirá una muestra de 20 ml de sangre para la medición de laboratorio que requiere que esté en ayunas ese día. Estas muestras se recolectarán en la clínica o en un centro de recolección local.

En esta visita se le proporcionará un reloj de actigrafía, que usted debe usar por un periodo de 7 días durante las 2 semanas después de la visita (post- visita). Este periodo de 7 días debiera incluir 2 días del fin de semana y 5 días de la semana . Ejemplo: de lunes a domingo. Este reloj registrará su actividad física y sueño y deberá ser devuelto prontamente al sitio de investigación (mediante correo certificado) para que su información pueda ser descargada.

Esta visita tomará aproximadamente entre 3 a 4 horas en completarse.

Las visitas del estudio se realizarán de manera presencial. Sin embargo, si hay circunstancias inesperadas que le impiden asistir a la clínica (por ejemplo, una pandemia), es posible que estas visitas se realicen de forma remota por videoconferencia. Si realiza estas visitas de forma remota, se le proporcionará un formulario para la toma de muestra en el laboratorio para que se tomen las muestras de sangre. No podrá participar en el subestudio si las visitas se realizan de forma totalmente remota.

4. ¿Cuáles son las alternativas para la participación?

La participación en este estudio de investigación es voluntaria. Puede haber otros tratamientos disponibles para manejar sus síntomas neurológicos prolongados causados por el COVID. Puede conversar sobre estas opciones con su médico de cabecera o con un especialista antes de decidir si es que quiere participar en este estudio, o no.

5. ¿Hay algún beneficio?

Este estudio apunta a generar conocimiento médico adicional y a mejorar el manejo de pacientes con síntomas neurológicos prolongados producto del COVID. Sin embargo, la participación en este estudio puede no beneficiarle directamente.

6. ¿Existen riesgos?

Todos los tratamientos médicos involucran algún riesgo de lesión o de efectos secundarios. Además, puede haber riesgos asociados con este estudio que, por el momento, son desconocidos o impredecibles. Los riesgos asociados con el medicamento del estudio que se está utilizando son bien conocidos y se describen a continuación.

Medicamento del estudio: atorvastatina

COMITÉ ÉTICO CIENTÍFICO
Facultad de Medicina
Clínica Alemana - Universidad del Desarrollo
Centro de Bioética
23.03.2023

.

La mayoría de las personas que toman medicamentos con estatinas las toleran muy bien. Sin embargo, algunas personas sufren efectos secundarios. Los efectos secundarios más comunes de las estatinas incluyen lo siguiente:

Efectos secundarios comunes- ocurren en 1 a 10 de cada 100 pacientes:

- Cefalea; dificultad para dormir; enrojecimiento de la piel; dolores musculares, dolor leve o debilidad muscular (mialgia); vahídos; mareos: náuseas o vómitos; calambres o dolores abdominales; hinchazón o gases; diarrea; estreñimiento; sarpullidos; bajos niveles de plaquetas en el torrente sanguíneo

Efectos secundarios poco comunes - ocurren en 1 a 10 de cada 1.000 pacientes:

- Nausea; pérdida del cabello; sensación de adormecimiento, pinchazos o cosquilleo en la piel; inflamación hepática, que puede causar síntomas similares a los de la gripe; inflamación pancreática, que pueda causar dolor abdominal; problemas a la piel, como sarpullido o acné; problemas sexuales, como disfunción eréctil y reducción de la libido.

Las estatinas también tienen advertencias de que la pérdida de memoria, confusión mental, neuropatías, altos niveles de azúcar en la sangre y diabetes tipo 2 son posibles efectos secundarios. Es importante recordar que las estatinas también pueden interactuar con otros medicamentos que usted tome.

Efectos secundarios raros y muy raros- ocurren en menos de 1 de cada 1.000 pacientes:

- Miositis, que es la inflamación de los músculos. El riesgo de lesiones musculares aumenta cuando se toman ciertos medicamentos junto con estatinas. Por ejemplo, si hay una combinación de una estatina con un fibrato, que es otro medicamento para reducir el colesterol, el riesgo de daños musculares aumenta significativamente si se compara con una persona que solo toma estatina.
- Niveles elevados de creatina quinasa (CK, por sus siglas en inglés), una enzima muscular que, cuando se eleva, puede causar dolores musculares, inflamación leve y debilidad muscular. Esta condición, aunque es poco común, puede tomar bastante tiempo en resolverse.
- Rabdomiólisis, inflamación y lesiones musculares extremas. Con esta condición, los músculos de todo el cuerpo duelen y se sienten débiles. Los músculos dañados severamente liberan proteínas al torrente sanguíneo, que luego son recolectadas por los riñones. Los riñones se pueden dañar al intentar eliminar una gran cantidad de músculo degradado debido al uso de estatinas. Esto puede, finalmente, llevar a una falla renal o incluso a la muerte. Afortunadamente, la rabdomiólisis es extremadamente rara. Ocurre en menos de una persona de cada 10.000 que toma estatinas.

Si usted experimenta cualquier dolor articular o muscular, dolor muscular leve o debilidad que no tenga explicación mientras está tomando el medicamento de la investigación, tendrá que contactarse con la oficina de investigación y ver a su médico lo antes que pueda.

<u>Recolección de muestras de sangre</u>

El riesgo de un examen de sangre incluye dolor, un moretón en el lugar desde donde se toma la muestra, rojez e inflamación de la vena, infección y, rara vez, desmayos.

<u>Riesgos reproductivos</u>

COMITÉ ÉTICO CIENTÍFICO
Facultad de Medicina
Clínica Alemana - Universidad del Desarrollo
Centro de Bioética
23.03.2023

.
Debido a que algunos estudios vinculan a las estatinas con malformaciones congénitas, se aconseja que quienes participen de este estudio no se embaracen. Es importante que las mujeres que participen del estudio no estén amamantando ni estén embarazadas, y que no se embaracen durante su participación en el estudio. Si usted es una mujer con la capacidad de quedar embarazada y hay alguna posibilidad de que esté embarazada, se le solicitará hacerse un test de embarazo. También se le solicitará usar un método anticonceptivo efectivo durante su participación en el estudio. Esto lo conversará con su médico al comienzo del estudio. Si, en cualquier momento durante su participación en el estudio, usted cree haber quedado embazada, es importante que notifique a su médico inmediatamente.

7. Compensación por lesiones o complicaciones

Es importante tener en cuenta que este es un estudio de bajo riesgo, pero en el caso de que usted sufriera cualquier lesión o complicaciones como resultado de este estudio, deberá contactar a su médico tan pronto sea posible, para que le ayude a obtener el tratamiento médico apropiado. Si usted está inscrito en Fonasa o Isapre, puede recibir el tratamiento médico requerido para su lesión o complicación como lo recibe habitualmente.

Además, puede tener el derecho de tomar acciones legales para obtener compensaciones por cualquier lesión o complicaciones que ocurrieran como resultado de este estudio. La compensación puede estar disponible si su lesión o complicación es lo suficientemente grave y es causada por drogas o equipamiento poco seguro, o por la negligencia de una de las partes involucradas en el estudio (por ej., el investigador o el médico tratante). Usted no renuncia a ningún derecho legal a obtener compensaciones al participar en este estudio.

8. ¿Tendrá la participación en este estudio un costo para mí, y se me pagará por participar?

Debido al tiempo que usted destinará a la participación en el estudio, se le reembolsarán $30.000 pesos en total por su participación en el proyecto, que se le pagarán una vez confirmados los criterios de selección. Además, se le entregarán $5.000 pesos por gastos de transporte en las visitas presenciales.

9. ¿Qué pasará con mis muestras?

La recolección de muestras de sangre durante este estudio de investigación es un componente obligatorio. Los exámenes se usan para determinar si es que el medicamento ha causado cualquier efecto secundario indeseado. Usted recibirá cualquier resultado que sea pertinente para su salud en general. Sin embargo, ninguna de las muestras se almacenará ni usará para investigaciones futuras. Todas las muestras se enviarán al laboratorio designado por el estudio para su análisis y, luego, serán desechadas, salvo que usted participe en el subestudio.

10. ¿Puedo tener otros tratamientos durante este proyecto de investigación?

Usted puede seguir con su tratamiento habitual mientras participa en este estudio. Sin embargo, no debería comenzar el estudio si tiene cualquier indicación o contraindicación sobre el uso de una estatina, que pueda afectar su salud. Si necesitara someterse a un tratamiento con estatina durante el transcurso del estudio, se le solicitará que deje de tomar el medicamento del estudio, pero que continúe siendo parte de él hasta la visita final, tal como se planificó. Si usted toma regularmente medicamentos de venta libre, por favor coménteselo al equipo del estudio.

COMITÉ ÉTICO CIENTÍFICO
Facultad de Medicina
Clínica Alemana - Universidad del Desarrollo
Centro de Bioética
23.03.2023

.
11. ¿Se podría detener este estudio de manera inesperada?

El estudio se podría detener de manera inesperada por varias razones, por ejemplo:

- efectos secundarios inaceptables o una decisión tomada por las autoridades sanitarias locales.

12. ¿Qué pasa si deseo retirarme de este proyecto de investigación?

Si usted decide retirarse del estudio, por favor notifique a algún miembro del equipo de investigación de inmediato e infórmele sobre cualquier problema médico que haya experimentado o sobre los medicamentos que haya tomado desde el contacto más reciente del estudio. Esto le permitirá al equipo de investigación discutir en mayor profundidad cualquier riesgo para la salud o requerimiento especial relacionado con su retiro del estudio.

Si usted retira su consentimiento para seguir con el tratamiento, los datos se seguirán recolectando, a menos que usted especifique lo contrario. Si decide dejar el estudio, los investigadores desearían mantener la información sobre su salud que se haya recolectado hasta ese momento. Esto es para ayudar a asegurarse de que los resultados de la investigación se puedan medir adecuadamente. Si decide retirarse y no desea que se use la información sobre su salud que ya se hubiera recolectado, por favor notifique al personal del estudio en el hospital al respecto.

13. ¿Qué ocurre cuando termina el proyecto de investigación?

Los medicamentos entregados durante este estudio no seguirán estando disponibles una vez terminado el estudio. Se le derivará de vuelta a su especialista o médico de cabecera para el manejo de sus síntomas neurológicos prolongados producto del COVID. Sin embargo, las estatinas como la atorvastatina no son costosas y están ampliamente disponibles. Su médico o especialista puede decidir si es adecuado que usted continúe tomando atorvastatina u otra estatina a largo plazo. Usted podrá entregar a su médico de cabecera los resultados de sus exámenes de sangre y los informes de las pruebas neuropsicológicas.

A veces, los estudios se extienden para que los investigadores puedan averiguar más sobre resultados en la salud a largo plazo. Es posible que el período de seguimiento de este estudio se extienda. Al firmar este formulario de consentimiento, usted está de acuerdo con que los investigadores le contacten en el futuro, para invitarle a participar en una fase de extensión del estudio.

14. Confidencialidad/privacidad

Cualquier información identificable que se recolecte sobre usted, en relación con este estudio, será confidencial y solo se revelará con su autorización, a menos que la ley requiera que ésta se revele. Solo los investigadores del estudio, los monitores, representantes de autoridades normativas y el comité de ética pueden tener acceso directo a ella. Este acceso se requiere para verificar la precisión de la información recolectada y asegurarse de que esta prueba que se está realizando cumple con los requerimientos y/o lineamientos normativos.

Los hallazgos clínicamente significativos que sean relevantes para su atención pueden ser compartidos con su médico de cabecera con su permiso. Si el asunto es urgente, es posible que debamos ponernos en contacto con su médico de cabecera en primera instancia para evitar retrasos.

COMITÉ ÉTICO CIENTÍFICO
Facultad de Medicina
Clínica Alemana - Universidad del Desarrollo
Centro de Bioética
23.03.2023

.

Estadísticos y académicos calificados, del The George Institute, o institutos de investigación académica similares, o universidades con los que The George Institute for Global Health colabore en Australia y en otros países, analizarán los datos.

Los monitores, auditores, representantes de las autoridades normativas y comités de ética también podrían recibir acceso directo a sus registros médicos originales, para la verificación de los procedimientos de la prueba y/o de los datos.

Toda la información transferida electrónicamente se almacenará en la base de datos primaria (IBM Clinical Development) y se hará una copia de seguridad en el servidor de IBM que se encuentra en los EE. UU., o se almacenará en una base de datos de investigación llamada REDCap. REDCap es una aplicación de base de datos segura basada en la web, respaldada en los servidores de Universidad del Desarrollo. Las videoconferencias se realizarán utilizando la plataforma Zoom. Se podrán realizar grabaciones cortas (1 minuto máximo) del proceso de consentimiento informado para tener mayor resguardo del mismo, con su autorización previa . Estos videos serán almacenados en una plataforma segura y con acceso protegido con clave. Toda la información se codificará para proteger su confidencialidad, y todos los registros computacionales serán protegidos mediante una contraseña. La documentación del estudio se mantendrá y archivará de manera segura por 15 años.

De acuerdo a las leyes australianas y del estado de Victoria sobre privacidad, y otras leyes pertinentes en Chile, usted tiene el derecho a solicitar que su información que haya sido recolectada y almacenada por el equipo de investigación. También tiene derecho a solicitar que cualquier información con la que no esté de acuerdo, sea corregida. Por favor, contacte al miembro del equipo del estudio nombrado al final de este documento si desearía tener acceso a su información.

15. Contribución a un banco de datos

Se buscará obtener la autorización del Comité de Ética para la Investigación en Seres Humanos (HREC, por sus siglas en inglés), antes de cualquier uso futuro de los datos. Aunque los estudios de investigación se establecen con un propósito principal, a menudo es útil para los científicos compartir la información que obtienen desde los estudios, con el fin de saber más sobre cómo se ve afectada la salud, y los tratamientos que funcionan mejor o peor en tipos particulares de pacientes de diferentes partes del mundo. Combinar la información de diferentes estudios en un solo lugar les ayuda a saber aún más acerca de la salud y el bienestar de las personas, y sobre la mejor forma de usar los tratamientos nuevos. La recolección de información a veces recibe el nombre de banco de datos. Deseamos almacenar los datos codificados de este estudio en uno o más de estos bancos de datos, donde junto con datos desde otros estudios, se puede usar para aumentar el conocimiento. Este trabajo podría ser realizado, o no, directamente por el personal de investigación asociado con este estudio, pero en la mayoría de los casos esto lo hace el personal que trabaja en los institutos de investigación o en las universidades. La información se incluirá en tales bancos de datos, de una manera en la que usted no pueda ser identificado. La ubicación de estos bancos de datos estará en el George Institute, o en institutos de investigación académica o universidades similares con los que The George Institute for Global Health colabora, en Australia y en otros países. No es posible determinar por cuánto tiempo se almacenarán los datos del estudio en cualquiera de los bancos de datos.

COMITÉ ÉTICO CIENTÍFICO
Facultad de Medicina
Clínica Alemana - Universidad del Desarrollo
Centro de Bioética
23.03.2023

.
El formulario de consentimiento para este estudio incluye una opción para que usted decida si desea, o no, que su información se use para los fines de este estudio, o si además entrega su consentimiento para que su información codificada se use para una investigación relacionada prolongada y, por lo tanto, consentiría a que su información se almacene en un banco de datos.

16. ¿Qué ocurre con los resultados?

Toda su información recolectada para este estudio se almacenará electrónicamente en una base de datos mantenida en una base de datos (IBM Clinical Development) y se respaldará en el servidor de IBM ubicado en los Estados Unidos . Se prevé que los resultados de este estudio se presenten o publiquen en conferencias médicas y en publicaciones científicas.

En cualquier publicación, la información se entregará de manera tal que usted no pueda ser identificado. Los resultados del estudio se le entregarán, si así lo desea. Al firmar este formulario de consentimiento, usted está de acuerdo con que sus datos se incluyan en los resultados publicados de este estudio.

Información adicional

Cuando haya leído esta información, el investigador principal o el coordinador de la investigación conversará con usted para responder cualquier pregunta que pudiera tener. Si usted quisiera saber más en cualquier etapa, por favor contacte al investigador principal o coordinador.

Aprobación ética
Toda investigación que involucre a seres humanos es revisada por un grupo independiente, llamado en Chile Comité Ético Científico. Este estudio ha sido aprobado por el Comité Ético Científico de la Facultad de Medicina Clínica Alemana - Universidad del Desarrollo, correo electrónico: ceccasudd@udd.cl

Información de contacto
Investigador principal en CAS UDD: Dra. Paula Muñoz
Teléfono de contacto: + 56225785561

Este estudio se realizará según lo dispuesto por las *Pautas Nacionales Sobre Conducta Ética en Investigación con Seres Humanos de Australia (2007, actualizadas en mayo de 2018)*. Estas pautas se desarrollaron para proteger los intereses de las personas que acuerdan participar en estudios de investigación con seres humanos.

Muchas gracias por tomarse el tiempo para considerar este estudio. Si desea participar de él, por favor firme el formulario de consentimiento adjunto. Este documento informativo es para que usted lo guarde.

COMITÉ ÉTICO CIENTÍFICO
Facultad de Medicina
Clínica Alemana - Universidad del Desarrollo
Centro de Bioética
23.03.2023

.

Consentimiento para participar en la investigación

**Título**	Tratamiento con estatina para el COVID-19, con el fin de optimizar la recuperación neurológica
**Título breve**	STRONGER
**Número de protocolo**	N/A
**Patrocinador del estudio**	The George Institute for Global Health
**Investigador responsable en CAS UDD**	Dra. Paula Muñoz

Yo, ____
[nombre]

he leído y comprendido la información para los participantes en el estudio de investigación nombrado anteriormente.

1. ____________________________________ ("el investigador") me ha informado sobre los procedimientos involucrados en el estudio, el tiempo involucrado en él, incluyendo cualquier inconveniencia, riesgos, incomodidades y potenciales efectos secundarios conocidos o esperados, y sus implicancias conocidas hasta el momento.

2. Entiendo que el investigador realizará el estudio de una manera que cumpla con los principios éticos y científicos establecidos por la Comisión Nacional de Salud e Investigación Médica (NHMRC, por sus siglas en inglés) de Australia y de Chile y las Pautas de Buenas Prácticas para la Investigación Clínica de la Administración de Bienes Terapéuticos.

3. Reconozco que se me ha dado el tiempo para considerar la información y para buscar consejos de otras fuentes.

4. Reconozco que rechazar a participar en este estudio no afectará el tratamiento usual de mi condición de salud.

5. Reconozco que me ofrezco de manera voluntaria para participar en este estudio y que me puedo retirar de él en cualquier momento.

6. Entiendo que mi participación en este estudio permitirá a los investigadores, y a otras personas, como se describe en la información para los participantes, tener acceso a mis antecedentes médicos, y estoy de acuerdo con esto.

7. Doy mi consentimiento para que mis datos de contacto se entreguen al servicio de correo certificado, para facilitar la entrega del medicamento del estudio.

COMITÉ ÉTICO CIENTÍFICO
Facultad de Medicina
Clínica Alemana - Universidad del Desarrollo
Centro de Bioética
23.03.2023

.

8. Entiendo que cualquier muestra de sangre recolectada solo se utilizará para este proyecto de investigación, según se describe en la parte pertinente del Documento Informativo para el Participante.

9. Reconozco que esta investigación ha sido aprobada por el Comité Ético Científico de la Facultad de Medicina Clínica Alemana - Universidad del Desarrollo

10. Reconozco que cualquier autoridad normativa puede tener acceso a mis antecedentes médicos relacionados con mi enfermedad y tratamiento, para los fines de este proyecto. Sin embargo, entiendo que mi identidad no será revelada a nadie más, ni en publicaciones o presentaciones.

11. Entiendo que se me puede contactar después del fin del estudio para que se me invite a participar de una evaluación posterior de mi salud y bienestar en el largo plazo.

12. Me gustaría recibir una copia de los resultados del estudio, una vez que estén disponibles. Mi dirección de correo electrónico es: ______________________________________

13. Entiendo que se me entregará una copia firmada de este documento y del Documento Informativo para el Participante, para que yo lo guarde.

Haga un círculo en *SÍ* o *NO* como respuesta a la siguiente afirmación

14. Estoy de acuerdo con que mi información recolectada durante este estudio también se incluya en uno o más bancos de datos, con el fin de extender la investigación, y entiendo que cualquiera de estos datos se almacenará de manera tal que no se me pueda identificar.

SÍ ***NO***

Nombre del participante ____

(letra imprenta - Nombre/Apellido)

Firma ______________________________ **Fecha** ____________________

COMITÉ ÉTICO CIENTÍFICO
Facultad de Medicina
Clínica Alemana - Universidad del Desarrollo
Centro de Bioética
23.03.2023

Facultad de Medicina
Clínica Alemana - Universidad del Desarrollo

stronger
Statin TReatment for COVID-19 to Optimise NeuroloGical recovERy

.

Declaración del representante del Director

Certifico el consentimiento del participante sobre su acuerdo de participar voluntariamente en este estudio de investigación.

**Nombre del Representante del Director**	______________________________ *(letra imprenta - Nombre/Apellido)*		
**Firma:**	______________________	**Fecha:**	______________

Declaración del Médico del Estudio/Investigador Senior [†]

He entregado una explicación verbal del proyecto de investigación, sus procedimientos y riesgos, y creo que el participante comprendió esa explicación.

**Nombre del Médico del Estudio/Investigador Senior [†]**	______________________________ *(letra imprenta - Nombre/Apellido)*		
**Firma**	______________________	**Fecha**	______________

[†] Un miembro senior del equipo de investigación debe entregar la explicación y la información relacionada con el proyecto de investigación.

Nota: todas las partes que firmen la sección sobre el consentimiento deben poner la fecha al lado de su propia firma.

COMITÉ ÉTICO CIENTÍFICO
Facultad de Medicina
Clínica Alemana - Universidad del Desarrollo
Centro de Bioética
23.03.2023

.

Forma de Retiro de Participación

**Título**	Tratamiento con estatina para el COVID-19, con el fin de optimizar la recuperación neurológica
**Título breve**	STRONGER
**Patrocinador del estudio**	The George Institute for Global Health, Sídney, Australia

Investigador Responsable en CAS-UDD Dra. Paula Muñoz

Declaración del Participante

Deseo retirar mi participación en el proyecto de investigación y entiendo que este retiro no afectará mi tratamiento de rutina o mi relación con los tratantes

Nombre del participante ______________________________
(letra imprenta)

Firma ______________________ **Fecha** ______________

Consentimiento otorgado para usar los datos recopilados hasta la fecha del retiro: Si ☐ No ☐

En el caso que la decisión de retiro del participante ha sido comunicada verbalmente, el médico/delegado/coordinador del estudio deberá entregar a continuación una descripción de las circunstancias:

Declaración del médico/coordinador del estudio:

He entregado una explicación verbal de las implicancias de retiro de este proyecto de investigación y creo que el participante ha entendido esta explicación

Nombre del médico/coordinador del estudio:______________________________
(letra imprenta)

Firma:______________________ Fecha:______________________

Nota: Todas las partes que firmen la sección de retiro deben colocar su propia firma

COMITÉ ÉTICO CIENTÍFICO
Facultad de Medicina
Clínica Alemana - Universidad del Desarrollo
Centro de Bioética
23.03.2023

Documento informativo y formulario de consentimiento para el participante

**Título**	Tratamiento con estatinas para el COVID-19 para optimizar la recuperación neurológica, sub estudio de biomarcador y resonancia magnética (STRONGER)
**Título breve**	Sub estudio STRONGER
**Número de protocolo**	N/A
**Patrocinador del estudio**	The George Institute for Global Health (TGI), Universidad de Nueva Gales del Sur, Sídney, Australia
**Investigador responsable en CAS-UDD**	Dra. Paula Muñoz

1. Introducción

Usted ha entregado su consentimiento para participar en el estudio de investigación que tiene como nombre "STRONGER". Este estudio apunta a determinar el efecto de utilizar una estatina (un medicamento para reducir el nivel de colesterol) para mejorar los efectos de síntomas "a largo plazo del COVID" en el cerebro. El tratamiento en este estudio involucra el uso de una estatina utilizada comúnmente, llamada atorvastatina. Como parte del estudio STRONGER, también invitamos a las personas a participar en el sub estudio STRONGER de biomarcador y resonancia magnética, para evaluar en mayor profundidad la efectividad del tratamiento en estudio, reduciendo cualquier señal de inflamación en el cerebro o sanguínea. Las imágenes por resonancia magnética (RM) se utilizarán para ver los marcadores de inflamación en el cerebro y se tomarán muestras adicionales de sangre, para ver los llamados biomarcadores neurodegenerativos.

El sub estudio STRONGER para biomarcador y resonancia magnética se está coordinando conjuntamente entre investigadores del George Institute for Global Health (TGI), de la Universidad de Nueva Gales del Sur, los departamentos de ingeniería eléctrica e informática, y de neurociencia (distrito hospitalario del Alfred Hospital) y la Monash University, en Melbourne, Australia.

2. Antecedentes y propósito del sub estudio STRONGER para biomarcador y resonancia magnética

Cada vez hay más evidencia que indica que los niveles bajos de inflamación en el cuerpo pueden mejorar la fuerza y el funcionamiento de los vasos sanguíneos, incluidos los del cerebro. Mediante nuevas tecnologías basadas en la resonancia magnética se pueden detectar los primeros signos de inflamación en el cerebro que provoca la liberación de proteínas a la sangre. Se ha demostrado que este tipo de anomalías ocurre con otras patologías cerebrales, incluso en personas con o en riesgo de padecer la enfermedad de Alzheimer

3. ¿Por qué se me ha seleccionado?

Se le ha solicitado participar en el subestudio del biomarcador y de resonancia magnética STRONGER porque usted, actualmente, está participando en el estudio STRONGER. Este subestudio se llevará a cabo en centros

COMITÉ ÉTICO CIENTÍFICO
Facultad de Medicina
Clínica Alemana - Universidad del Desarrollo
Centro de Bioética
23.03.2023

médicos seleccionados en Australia y en Chile, donde se realizará un tipo de resonancia magnética específica. Se invitará a aproximadamente 220 personas a participar.

4. ¿Qué involucrará mi participación en el sub estudio del biomarcador y de resonancia magnética?

Participar involucra tomarse dos scanner con equipo de resonancia magnética. Esto se realizará en los siguientes momentos:

1. Visita 2, ya sea el mismo día o dentro de los siguientes 7 a 10 días desde la evaluación clínica de investigación de antecedentes/de referencia (dependiendo de la disponibilidad en la agenda).
2. Visita 5, en la visita final del estudio, 12 meses (± 1 mes) desde el comienzo del estudio

Se le pedirá entregar una muestra de sangre de 28.5 ml (nota: la cantidad total de sangre necesaria para el estudio principal y este sub estudio es de 48.5 ml) para la medición del laboratorio. Las muestras de sangre se deberían tomar en la mañana.

Cada RM tomará aproximadamente 45 minutos en completarse. Las citas para sus RM las agendará el coordinador del estudio STRONGER/miembro del staff, para el momento que mejor le acomode. Las RM se realizarán en Clínica Alemana.

Si está de acuerdo en participar en este estudio, se le pedirá firmar el Formulario de Consentimiento para el Participante, para confirmar que usted comprende el propósito y lo que involucra el sub estudio de biomarcador y resonancia magnética STRONGER y que usted tiene la libertad de abandonarlo en cualquier momento.

Se le notificará oportunamente si hay información disponible que pudiera ser relevante para su voluntad de continuar su participación en el subestudio de biomarcador y resonancia magnética STRONGER.

5. ¿Cuáles son las alternativas para la participación?

La participación en este estudio de investigación es voluntaria. Puede conversar con su médico de cabecera o con un especialista antes de decidir si es que quiere participar en este estudio o no.

6. ¿Hay algún beneficio?

El subestudio apunta a generar confirmación médica adicional y a mejorar el manejo de pacientes con COVID-19 prolongado. Sin embargo, la participación en el subestudio de biomarcador y resonancia magnética podría no beneficiarle directamente.

7. ¿Existen riesgos?

Todos los tratamientos médicos involucran algún riesgo de lesión o de efectos secundarios. Además, puede haber riesgos asociados con el subestudio de biomarcador y resonancia magnética STRONGER que, por el momento, son desconocidos o impredecibles. Los riesgos asociados con los exámenes de resonancia magnética son bien conocidos y se describen a continuación.

Los exámenes de resonancia magnética pueden causar una ansiedad leve. Sin embargo, esto usualmente se reduce cuando comienza el examen, y los investigadores están entrenados para lidiar con estas situaciones. Si usted es claustrofóbico(a), es decir, que sufre de un miedo extremo a los espacios cerrados, puede no ser

COMITÉ ÉTICO CIENTÍFICO
Facultad de Medicina
Clínica Alemana - Universidad del Desarrollo
Centro de Bioética
23.03.2023

apropiado que se realice un examen de resonancia magnética. A veces, se puede usar un sedante suave para que se relaje. Esto podría implicar que tenemos que reagendar su visita para la resonancia magnética si decide proceder con esta investigación.
Los exámenes de resonancia magnética involucran a un campo magnético fuerte y estático, que puede ocasionar las siguientes preocupaciones de seguridad:

- el campo atraerá a objetos magnéticos (desde elementos pequeños como llaves y teléfonos celulares, un marcapasos o una prótesis de cadera, a elementos grandes y pesados, como tanques de oxígeno), que puede hacer que estos objetos se conviertan en proyectiles. Al revisar cuidadosamente a las personas y retirar cualquier objeto metálico antes de ingresar al ambiente de la resonancia magnética y al campo magnética, se minimizará este riesgo. Usted no pasará por una resonancia magnética si tiene un implante metálico.
- durante la resonancia magnética, escuchará ruidos fuertes, parecidos a golpeteos. Estos ruidos pueden dañar la audición si no se usa la protección auditiva adecuada. Esta protección se entrega para minimizar ese riesgo.
- la energía de radiofrecuencia usada durante el scanner de resonancia magnética puede llevar a un calentamiento leve del cuerpo. El potencial para este calentamiento es mayor durante resonancias magnéticas de larga duración, y es menos probable durante un scanner de 45 minutos, planificado para este sub estudio.

Recolección de muestras de sangre
El riesgo de un examen de sangre incluye dolor, un moretón en el lugar desde donde se toma la muestra, rojez e inflamación de la vena, infección y, rara vez, desmayos.

8. ¿Qué ocurre si el examen de resonancia magnética revela una anormalidad de la que yo no tenía conocimiento?
Los procedimientos usados en este estudio no son diagnósticos. Sin embargo, los exámenes de resonancia magnética podrían, incidentalmente, revelar una anormalidad inesperada. Todos los exámenes de resonancia magnética son revisados por un radiólogo clínico, y cualquier anormalidad se reporta. El radiólogo clínico entregará un informe sobre la anormalidad, incluyendo recomendaciones sobre si es necesario realizar un seguimiento clínico.

Después de su examen, las imágenes serán enviadas a los especialistas de la investigación ubicados en la Monash University en Melbourne, Australia, quienes las examinarán para realizar mediciones y para detectar cualquier anormalidad. Estas pruebas para la inflamación cerebral no se realizan rutinariamente durante un examen clínico y no están ampliamente disponibles para los pacientes como parte de la práctica normal. Esto no se realizará el día de su examen. Usted debería saber que, debido a que las imágenes se toman para los fines específicos de la investigación, no todas las anormalidades que podrían ser detectadas en otro examen de resonancia magnética se verán, necesariamente.

En el caso poco probable de que se encuentre una anormalidad en su resonancia magnética cerebral para la que se recomiende tratamiento o seguimiento, u otra información relevante para su salud, le enviaremos un informe para que lo entregue a su médico de cabecera. También le contactaremos para entregarle asesoría adicional sobre los pasos a seguir.

COMITÉ ÉTICO CIENTÍFICO
Facultad de Medicina
Clínica Alemana - Universidad del Desarrollo
Centro de Bioética
23.03.2023

9. Compensación por lesiones o complicaciones

Es importante tener en cuenta que este es un estudio de bajo riesgo, pero en el caso de que usted sufriera cualquier lesión o complicaciones como resultado del sub estudio de biomarcador y resonancia magnética STRONGER, deberá contactar a su médico tan pronto sea posible, para que le ayude a obtener el tratamiento médico apropiado. Si usted está inscrito en Fonasa o Isapre, puede recibir el tratamiento médico requerido para su lesión o complicación como lo recibe habitualmente.

Además, puede tener el derecho a tomar acciones legales para obtener compensaciones por cualquier lesión o complicaciones que ocurrieran como resultado del subestudio de biomarcador y resonancia magnética STRONGER. La compensación puede estar disponible si su lesión o complicación es lo suficientemente grave y es causada por drogas o equipamiento poco seguro, o por la negligencia de una de las partes involucradas en el sub estudio de biomarcador y resonancia magnética STRONGER (por ej., el investigador o el médico tratante). Usted no renuncia a ningún derecho legal a obtener compensaciones al participar en este subestudio de biomarcador y resonancia magnética STRONGER. Si usted es elegible para tratamiento médico, puede recibirlo para su lesión o complicación como se trata habitualmente.

10. ¿Me costará algo participar en el estudio?

La participación en el sub estudio de biomarcador y resonancia magnética no le costará nada. Sin embargo, se le reembolsarán los gastos de transporte razonable para asistir al centro de RM, si es que este está en una ubicación diferente al lugar del estudio.

11. ¿Qué ocurrirá con mis muestras de sangre y mis imágenes de resonancia magnética?

Las muestras de sangre se recolectarán en la clínica a la que asiste; las muestras se procesarán y almacenarán en este lugar hasta el final del estudio. Luego, será enviada a los departamentos de ingeniería eléctrica e informática, y de neurociencia (distrito hospitalario del Alfred Hospital) y a la Monash University en Melbourne, Australia, donde sus muestras de sangre se analizarán en busca de biomarcadores de inflamación. Estas muestras se almacenarán para análisis exploratorios futuros y solo con el fin de examinar la inflamación y degeneración del cerebro.

Sus exámenes de resonancia magnética codificados se registrarán en un computador y los datos sin identificación se almacenarán en un servidor en la Monash University en Melbourne, Australia. Su sitio de estudio también almacenará una copia de su examen. Todos las RM se almacenarán por al menos 15 años después de finalizado el subestudio de biomarcador y resonancia magnética STRONGER.

12. ¿Puedo tener otros tratamientos mientras participo en el subestudio de biomarcador y resonancia magnética STRONGER?

Usted puede continuar con sus tratamientos habituales a lo largo del sub estudio de biomarcador y resonancia magnética. Sin embargo, si llegara a necesitar el implante de cualquier dispositivo metálico, usted debe notificar a su médico del estudio STRONGER y debe retirarse de la participación futura en el subestudio de biomarcador y resonancia magnética STRONGER.

COMITÉ ÉTICO CIENTÍFICO
Facultad de Medicina
Clínica Alemana - Universidad del Desarrollo
Centro de Bioética
23.03.2023

13. ¿Qué pasa si deseo retirarme del proyecto de investigación?

La participación en este subestudio es voluntaria. Si no desea participar, no tiene que hacerlo. Si decide participar y, más adelante, cambia de opinión, es libre de retirarse del proyecto en cualquier momento.

Si usted decide retirarse del subestudio, por favor notifique a algún miembro del equipo de investigación de inmediato e infórmele sobre cualquier problema médico que haya experimentado o sobre los medicamentos que haya tomado desde el contacto más reciente del estudio. Esto le permitirá al equipo de investigación discutir en mayor profundidad cualquier riesgo para la salud o requerimiento especial relacionado con su retiro del estudio.

14. ¿Qué ocurre cuando el sub estudio de biomarcador y resonancia magnética STRONGER termine?

Cuando se complete el subestudio de biomarcador y resonancia magnética STRONGER, quedarán disponibles copias de las RM para usted o para su médico tratante/de cabecera, si así lo solicita.

15. Confidencialidad/privacidad

Cualquier información identificable que se recolecte sobre usted, en relación con este subestudio de biomarcador y resonancia magnética STRONGER, será confidencial y solo se revelará con su autorización, a menos que la ley requiera que esta se revele. Solo los investigadores del estudio STRONGER y del subestudio STRONGER, los monitores, representantes de las autoridades normativas pertinentes y los comités de ética tendrán acceso directo a ella. El acceso se requiere para verificar la precisión de la información recolectada y la asegurarse de que esta prueba se está realizando cumpliendo con los requerimientos y/o con los lineamientos normativos.

Estadísticos y académicos calificados, del George Institute, o institutos de investigación académica similares, o universidades con los que el George Institute for Global Health colabore en Australia y en otros países, analizarán los datos.

Los monitores del estudio STRONGER, auditores, representantes de las autoridades normativas y comités de ética también podrían recibir acceso directo a sus registros médicos originales, para la verificación de los procedimientos de la prueba y/o de los datos.

Toda la información transferida de manera electrónica se almacenará en una base de datos (IBM Clinical Development) y se respaldará en el servidor de IBM ubicado en los Estados Unidos, o se almacenará en una base de datos llamada REDCap. REDCap es una aplicación de base de datos segura basada en la web, alojada y respaldada en los servidores de Universidad del Desarrollo. Toda la información se codificará para proteger su confidencialidad y todos los registros de la computadora estarán protegidos con contraseña. La documentación de prueba se conservará y archivará de forma segura durante 15 años

La información del sub estudio de biomarcador y resonancia magnética STRONGER podría ser recolectada por investigadores entrenados, designados por el George Institute, quienes trabajarán de manera independiente a quienes manejan el sub estudio de biomarcador y resonancia magnética STRONGER. Al firmar este formulario de consentimiento, usted demuestra estar de acuerdo con que sus detalles de contacto se almacenen en una base de datos protegida con contraseña, a la que solo podrá acceder el personal del hospital y los investigadores

COMITÉ ÉTICO CIENTÍFICO
Facultad de Medicina
Clínica Alemana - Universidad del Desarrollo
Centro de Bioética
23.03.2023

independientes designados, quienes podrían contactarle para saber si ha sufrido de alguna enfermedad grave o si ha sido hospitalizado durante el estudio. Esta base de datos será independiente de la base de datos que contiene sus datos del sub estudio de biomarcador y resonancia magnética STRONGER, y estas no estarán vinculadas de ninguna manera, para que la confidencialidad y la imposibilidad de que se le identifique con la información recolectada sobre usted, para los fines del sub estudio de biomarcador y resonancia magnética STRONGER, se mantengan.

16. ¿Qué ocurre con los resultados?

Toda la información recolectada sobre usted en el sub estudio de biomarcador y resonancia magnética STRONGER se almacenará electrónicamente en bases de datos mantenidas por el George Institute y los Departamentos de ingeniería eléctrica e informática, y de neurociencia (distrito hospitalario del Alfred Hospital), y la Monash University, en Melbourne, Australia. Se prevé que los resultados del subestudio de biomarcador y resonancia magnética STRONGER se presenten o publiquen en conferencias médicas y en publicaciones científicas. En cualquier publicación, la información se entregará de manera tal que usted no pueda ser identificado. Los resultados del subestudio de biomarcador y resonancia magnética STRONGER se le entregarán, si así lo desea. Al firmar este formulario de consentimiento, usted está de acuerdo con que sus datos se incluyan en los resultados publicados del subestudio de biomarcador y resonancia magnética STRONGER.

17. Información adicional

Cuando haya leído esta información, el investigador principal o el coordinador de la investigación conversará con usted para responder cualquier pregunta que pudiera tener. Si usted quisiera saber más en cualquier etapa, por favor contacte al investigador principal o coordinador.

Aprobación ética

Toda investigación que involucre a seres humanos es revisada por un grupo independiente, llamado en Chile Comité Ético Científico. Este estudio ha sido aprobado por el Comité Ético Científico de la Facultad de Medicina Clínica Alemana - Universidad del Desarrollo, correo electrónico: ceccasudd@udd.cl

Información de contacto
Investigador principal: Dra. Paula Muñoz
Número de contacto: + 56225785591

Muchas gracias por tomarse el tiempo para considerar el subestudio de biomarcador y resonancia magnética STRONGER. Si desea participar de él, por favor firme el formulario de consentimiento adjunto. Este documento informativo es para que usted lo guarde.

COMITÉ ÉTICO CIENTÍFICO
Facultad de Medicina
Clínica Alemana - Universidad del Desarrollo
Centro de Bioética
23.03.2023

Consentimiento para participar en la investigación

**Título**	Tratamiento con estatinas para el COVID-19 para optimizar la recuperación neurológica, sub estudio de biomarcador y resonancia magnética (STRONGER)
**Título breve**	STRONGER
**Número de protocolo**	N/A
**Patrocinador del proyecto**	George Institute for Global Health (TGI), Universidad de Nueva Gales del Sur, Sídney, Australia
**Investigador responsable en CAS-UDD**	Dra. Paula Muñoz

Yo, ____
[nombre]

he leído y comprendido la información para los participantes en el sub estudio de biomarcador y resonancia magnética STRONGER nombrado anteriormente.

1. __________________________________ ("el investigador") me ha informado sobre los procedimientos involucrados en el sub estudio de biomarcador y resonancia magnética STRONGER, el tiempo involucrado en él, incluyendo cualquier inconveniencia, riesgos, incomodidades y potenciales efectos secundarios conocidos o esperados, y sus implicancias conocidas hasta el momento.

2. Entiendo que el investigador realizará el sub estudio de biomarcador y resonancia magnética STRONGER de una manera que cumpla con los principios éticos y científicos establecidos por la Comisión Nacional de Salud e Investigación Médica (NHMRC, por sus siglas en inglés) de Australia y las Pautas de Buenas Prácticas para la Investigación Clínica de la Administración de Bienes Terapéuticos.

3. Reconozco que se me ha dado el tiempo para considerar la información y para buscar consejos de otras fuentes.

4. Reconozco que rechazar mi participación en el subestudio de biomarcador y resonancia magnética STRONGER no afectará el tratamiento habitual de mi condición o la continuación de mi participación en el estudio STRONGER.

5. Reconozco que me ofrezco de manera voluntaria para participar en este subestudio de biomarcador y resonancia magnética STRONGER y que me puedo retirar de él en cualquier momento.

COMITÉ ÉTICO CIENTÍFICO
Facultad de Medicina
Clínica Alemana - Universidad del Desarrollo
Centro de Bioética
23.03.2023

6. Entiendo que cualquier examen de resonancia magnética y muestras de sangre recolectadas solo se usarán para el sub estudio de biomarcador y resonancia magnética STRONGER, como se describe en la sección pertinente del Documento Informativo para Participantes.

7. Reconozco que el sub estudio de biomarcador y resonancia magnética STRONGER ha sido aprobado por el Comité Ético Científico de la Facultad de Medicina Clínica Alemana - Universidad del Desarrollo

8. Reconozco que cualquier autoridad normativa puede tener acceso a mis antecedentes médicos relacionados con mi enfermedad y tratamiento, para los fines del sub estudio de biomarcador y resonancia magnética STRONGER. Sin embargo, entiendo que mi identidad no será revelada a nadie más, ni en publicaciones o presentaciones.

9. Entiendo que se me entregará una copia firmada de este documento y del Documento Informativo para el Participante, para que yo lo guarde.

**Nombre del participante**	______________________________ *(letra imprenta - Nombre/Apellido)*		
**Firma**	______________________	**Fecha**	______________

Declaración del representante del Director

Certifico el consentimiento del participante, que acuerda voluntariamente participar en el subestudio de biomarcador y resonancia magnética STRONGER.

**Nombre del representante del Director**	______________________________ *(letra imprenta)*		
**Firma**	______________________	**Fecha**	______________

Declaración del Médico del Estudio/Investigador Senior †

He entregado una explicación verbal del sub estudio de biomarcador y de resonancia magnética STRONGER, sus procedimientos y riesgos, y creo que el participante comprendió esa explicación.

**Nombre del Médico del Estudio/Investigador Senior**	______________________________		
**Firma**	______________________	**Fecha**	______________

Nota: todas las partes que firmen la sección sobre el consentimiento deben poner la fecha al lado de su propia firma.

COMITÉ ÉTICO CIENTÍFICO
Facultad de Medicina
Clínica Alemana - Universidad del Desarrollo
Centro de Bioética
23.03.2023

Forma de Retiro de Participación

**Título**	Tratamiento con estatina para el COVID-19, con el fin de optimizar la recuperación neurológica
**Título breve**	STRONGER
**Patrocinador del estudio**	The George Institute for Global Health, Sidney, Australia

Investigador Responsable en CAS-UDD Dra. Paula Muñoz

Declaración del Participante

Deseo retirar mi participación en el proyecto de investigación y entiendo que este retiro no afectará mi tratamiento de rutina o mi relación con los tratantes

Nombre del participante ______________________________
(letra imprenta)

Firma ______________________ **Fecha** ______________

Consentimiento otorgado para usar los datos recopilados hasta la fecha del retiro: Si ☐ No ☐

En el caso que la decisión de retiro del participante ha sido comunicada verbalmente, el médico/delegado/coordinador del estudio deberá entregar una descripción de las circunstancias a continuación:

Declaración del médico/coordinador del estudio:

He entregado una explicación verbal de las implicancias de retiro de este proyecto de investigación y creo que el participante ha entendido esta explicación

Nombre del médico/coordinador del estudio:_ ______________________________
(letra imprenta)

Firma:______________________ **Fecha:**______________________

Nota: Todas las partes que firmen la sección de retiro deben colocar su propia firma

COMITÉ ÉTICO CIENTÍFICO Facultad de Medicina Clínica Alemana - Universidad del Desarrollo Centro de Bioética 23.03.2023
