# Supplementary material for: STatin TReatment for COVID-19 to Optimise NeuroloGical recovERy (STRONGER): study protocol for a randomised, open label clinical trial in patients with persistent neurological symptoms after COVID-19 infection
Source: BMJ Open. 2025 Apr 14;15(4):e089382. doi: 10.1136/bmjopen-2024-089382 (PMC11997840; doi:10.1136/bmjopen-2024-089382)
Supplement: online supplemental file 1 [file bmjopen-15-4-s001.pdf]

ADDRESS FOR ALL CORRESPONDENCE
RESEARCH ETHICS AND GOVERNANCE OFFICE
ROYAL PRINCE ALFRED HOSPITAL
CAMPERDOWN NSW 2050
TELEPHONE: (02) 9515 6766
EMAIL: SLHD-RPAEthics@health.nsw.gov.au
REFERENCE: **X21-0113 & 2021/ETH00777**
10.15/MAY21

NSW GOVERNMENT | Health Sydney Local Health District

19 May 2021

This letter constitutes ethical approval only. You must NOT commence this research project at ANY site until you have submitted a Site Specific Assessment Form to the Research Governance Officer and received separate authorisation from the Chief Executive or delegate of that site.

Dear Professor Anderson,

Re: Protocol No X21-0113 & 2021/ETH00777 - "Statin TReatment for COVID-19 to Optimise NeuroloGical recovery"

The Executive of the Ethics Review Committee, at its meeting of 18 May 2021 considered your correspondence of 12 May 2021. In accordance with the decision made by the Ethics Review Committee at its meeting of 26 April 2021, ethical approval is granted*.

- The research project meets the requirements of the *National Statement on Ethical Conduct in Human Research*.

This approval includes the following:

- HREA (Version 2, 28 April 2021)
- Protocol (Version 2.0, 12 May 2021)
- Participant Information Sheet/Consent Form (Master Version 2.0, 5 May 2021)
- Participant Information Sheet/Consent Form - Biomarker & MRI Sub-Study (Master Version 2.0, 5 May 2021)
- Telephone Script Form C – 6 months (Version 1.0, 6 May 2021)
- Telephone Script Form E – 12 months (Version 1.0, 6 May 2021)
- SAE AESI CRF Telephone Script (Version 1.0, 6 May 2021)
- GeneActiv Instructions 1 week (Version 1.0, 6 May 2021)
- 7-day Sleep Diary (Version 1.0, 6 May 2021)
- Remote Consent GP Letter (Version 1.0, 4 May 2021)

- Screen Failure GP letter (Version 1.0, 4 May 2021)
- Randomisation GP Letter (Version 1.0, 4 May 2021)
- Lost to Follow-Up GP Letter (Version 1.0, 4 May 2021)
- Withdrawal GP Letter (Version 1.0, 4 May 2021)
- Study Completion GP Letter (Version 1.0, 4 May 2021)
- Research Data Management Plan (Version 1.0, 10 March 2021)
- Victorian Specific Module (6 May 2021)

***Additional conditions of this approval:**

- Approval from the RPA Virtual Head of Department must be sought for the RPAH Site Specific Assessment
- All advertising material for use must be submitted to the HREC for review and approval as soon as they become available.

You are asked to note the following:

- **This study requires notification to the Therapeutic Goods Administration (TGA) under the Clinical Trials Notification (CTN) Scheme.**
- The Committee noted that authorisation will be sought to conduct the study at the following sites:
    - Royal Prince Alfred Hospital, NSW
    - The Brain and Mind Centre of the University of Sydney, NSW
    - The Alfred, VIC
    - Monash University, VIC
- It is a requirement of ethics approval that, before its commencement, this clinical trial is registered on a publicly accessible register, such as the Australian New Zealand Clinical Trials Registry or another appropriate international register. The Committee therefore sought details of the Register in which the study has been included and its registration number.
- This approval is valid for five years, and the Committee requires that you furnish it with **annual reports** on the study's progress beginning in **May 2022.** If recruitment is ongoing at the conclusion of the four year approval period, a full re-submission will be required. Ethics approval will continue during the re-approval process.
- This human research ethics committee (HREC) has been accredited by the NSW Department of Health as a lead HREC under the model for single ethical and scientific review and is constituted and operates in accordance with the National Health and Medical Research Council's *National Statement on Ethical Conduct in Human Research* and the *CPMP/ICH Note for Guidance on Good Clinical Practice*.

- You must immediately report anything which might warrant review of ethical approval of the project in the specified format, including unforeseen events that might affect continued ethical acceptability of the project.

- You must notify the HREC of proposed changes to the research protocol or conduct of the research in the specified format.

- You must notify the HREC and other participating sites, giving reasons, if the project is discontinued at a site before the expected date of completion.

- If you or any of your co-investigators are University of Sydney employees or have a conjoint appointment, you are responsible for informing the University's Risk Management Office of this approval, so that you can be appropriately indemnified.

- Where appropriate, the Committee recommends that you consult with your Medical Defence Union to ensure that you are adequately covered for the purposes of conducting this study.

Should you have any queries about the Committee's consideration of your project, please contact me. The Committee's Terms of Reference, Standard Operating Procedures, membership and standard forms are available from the Sydney Local Health District website.

If you are not using REGIS, a copy of this letter must be forwarded to all site investigators for submission to the relevant Research Governance Officer.

The Ethics Review Committee wishes you every success in your research.

Regards,

S. Thomas

Sanaa Thomas
Executive Officer
Clinical Trials Sub-committee (RPAH Zone)

for:

Merela Ghazal
A/Executive Officer
Ethics Review Committee (RPAH Zone)

HERC\EXCOR\21-05

COMITÉ ÉTICO CIENTÍFICO
FACULTAD DE MEDICINA
CLÍNICA ALEMANA DE SANTIAGO
UNIVERSIDAD DEL DESARROLLO

Facultad de Medicina
Clínica Alemana - Universidad del Desarrollo

ACTA DE APROBACIÓN
2021-75

Fecha: **Santiago, 20 de agosto 2021**
Protocolo: **"Statin Treatment for Covid-19 to Optimise Neurological Recovery" (STRONGER) N° UIEC 1074**
Investigador Responsable: **Paula Muñoz**
Institución: **Departamento de Psiquiatría y Neurología**
Clínica Alemana de Santiago

Los siguientes documentos han sido analizados a la luz de los postulados de la Declaración de Helsinski, de las Pautas éticas internacionales para la investigación relacionada con la salud con seres humanos que involucra sujetos humanos CIOMS 2016, y de las Guías de Buena Práctica Clínica de ICH 1996.

Documentación revisada en primera instancia:

1. Protocolo del estudio, versión 2.0 con fecha 12 de mayo de 2021, en inglés (incluye resumen en español)
2. Consentimiento informado para el participante, versión local 1.0 con fecha 17 de julio de 2021, en español.
3. Consentimiento informado para la realización de sub-estudio de biomarcardores y resonancia magnética, versión 1.0 con fecha 02 de julio de 2021, en español
4. Folleto de información para el profesional, ISP 24 octubre 2012
5. Guía de instrucciones para el participante del uso del actígrafo, versión 1.0 del 06 de mayo 2021, en español
6. Diario de sueño para el participante, versión 1.0 del 06 de mayo 2021, en español
7. Currículum vitae del investigador responsable para Clínica Alemana y coinvestigadores (Dra. Ximena Stecher, Dr. Carlos Delfino y Ps. Helga Welzel) y coordinador local (Flga. Francisca Urrutia).
8. Certificados de Buenas Prácticas Clínicas del equipo
9. Declaración de conflictos de interés del equipo
10. Carta de apoyo de Jefes de Departamento de Neurología y Psiquiatría y de Servicio de Neurología de Clínica Alemana
11. Material para difusión
12. Seguro está pendiente de envío

COMITÉ ÉTICO CIENTÍFICO
Facultad de Medicina

Documentación revisada en respuesta a carta de observaciones de fecha 02 de agosto de 2021:

1. Carta respuesta de fecha 15 de agosto 2021
2. Consentimiento informado para el participante, versión local 1.0 de fecha 15 agosto 2021
3. Consentimiento informado para la realización de sub-estudio de biomarcardores y resonancia magnética, versión 1.0 de fecha 15 agosto 2021
4. Material para difusión: poster A4 y A5 enviados con las modificaciones solicitadas

Para otorgar la presente Acta de Aprobación, se consideraron los siguientes aspectos éticos:

1.- Valor social: este es un estudio internacional, Fase III, a realizarse en Chile y Australia, cuyo objetivo es determinar si 40 mg de atorvastatina diaria mejora la función neurocognitiva en adultos con síntomas neurológicos post COVID. La importancia radica en que se ha hecho evidente que un grupo significativo de pacientes experimenta síntomas neurológicos post COVID-19, que perduran en el tiempo, en lo que ha sido llamado COVID-largo, en el cual se postula existe un componente inflamatorio. Es en este posible mecanismo donde podrían actuar las estatinas, compuestos que mediante la inhibición de mevalonate tienen un "essential role for intracellular inflammatory signalling and pro-inflammatory cytokine responses". Asimismo, ya existe evidencia que el uso de estatinas previene el deterioro cognitivo.

2.- Validez Científica: es estudio intervencional, randomizado, abierto, ciego al evaluador, que compara dos intervenciones: uso de atorvastatina 40 mg/día vs tratamiento estándar por 18 meses, en un grupo de 410 adultos que tuvieron COVID. Tiene sub-estudio para futuros estudios de biomarcadores neurodegenerativos y resonancia magnética cerebral (n = 220).

Dentro de los estudios experimentales existen diferentes tipos de diseños que son apropiados para un ensayo clínico en fase 3. Si bien el *gold standard* son los estudios randomizados contra placebo, en este caso, el tipo de estudio planteado permite responder la pregunta de investigación mediante las evaluaciones de resultados cognitivos (evaluación ciega del desenlace por profesionales expertos) y biomarcadores (estos últimos, marcadores objetivos, por lo que no varían según la interpretación humana). Además, se contempla una adjudicación ciega de los eventos adversos por ramas, que serán considerados en los resultados finales. Los sesgos que pueden asociarse la metodología de un estudio abierto serán entonces minimizados con las evaluaciones objetivas y enmascaradas que fueron mencionadas previamente. Actualmente, y sobre todo debido a la pandemia COVID-19, este tipo de estudios están siendo utilizados, ya que es una forma más rápida y eficaz de brindar conocimiento científico de calidad ante situaciones como las que debemos enfrentar como sociedad en una emergencia sanitaria

3.- Evaluación riesgo/beneficio: favorable. El uso de atorvastatina es de bajo riesgo, toda vez que es fármaco muy usado y donde existe abundante experiencia clínica. La RNM no requiere de medio de contraste**.** Los tests cognitivos no son riesgosos. Con respecto a los resultados de marcadores de neurodegeneración, en el consentimiento informado se aclara que dichos procedimientos no son diagnósticos, por lo que no tendrán relevancia clínica para el paciente ni sus tratantes. Además, el procesamiento de las muestras se realizará en la última etapa del estudio.

4.- Conflictos de interés: no se observan fueran del indudable interés académico.

5.- Consentimiento informado: contempla firma del consentimiento informado por medios electrónicos y presenciales. Se entregará un incentivo a cada participante del estudio de $30.000 en

total, el cual se realizará una vez corroborados los criterios de inclusión y luego de la randomización. Este pago, tal como se manifiesta en la carta, es planteado como reembolso de gastos (se estiman 220 participantes en el estudio).

Con respecto a las muestras de sangre, el patrocinador del estudio (George Institute) aclaró que las muestras de sangre no serán transferidas a terceros u otros países. La información que podría ser compartida serán solo los datos del estudio. En el caso de que se pretendan utilizar estos datos en el futuro, se solicitará la aprobación del Comité Ético científico antes de cualquier nuevo uso, tal como se describe en el formulario de consentimiento.

6.- Protección de los derechos de los participantes: es adecuada. Hay libertad de participación, derecho a retiro, se resguardará la confidencialidad, y no hay costos asociados a los participantes.

Por lo tanto, el Comité de Ética considera que el estudio está bien justificado, que no hay objeciones éticas para la realización del estudio propuesto, y, por ende, otorga su aprobación, la que estará vigente mientras el proyecto esté en curso.

El Comité solicita que el investigador:

- Utilice los documentos de consentimiento informado versión 1.0 de fecha 15 de agosto, aprobados y timbrados por este Comité, así como el material para difusión.
- Para nuevas correspondencias referentes a este proyecto, se le solicita utilizar el número **2021-75**.
- Una vez finalizado el proyecto, el Comité deberá ser informado de los resultados del estudio.

Dr. Marcial Osorio
Presidente
Comité Ético Científico

Javiera Bellolio A.
Secretaria Ejecutiva
Comité Ético Científico

COMITÉ ÉTICO CIENTÍFICO
UDD
Facultad de Medicina

Nómina de los integrantes del Comité Ético Científico de la Facultad de Medicina Clínica Alemana Universidad del Desarrollo que participaron de la reunión de fecha 29 de julio de 2021, donde se acordó la aprobación del protocolo una vez subsanadas las observaciones:

NOMBRE	PROFESIÓN	CARGO
Marcial Osorio Fuenzalida	Médico	Presidente
Bernardita Portales V.	Fonoaudióloga	Vicepresidenta
Javiera Bellolio Avaria	Abogado	Secretaria Ejecutiva
Alejandra Valdés Valdés	Matrona	Integrante
Juan Alberto Lecaros Urzúa	Abogado	Integrante
Sofía Salas Ibarra	Médico	Integrante
Andrea Schilling Redlich	Médico	Integrante
Gonzalo López Gaete	Abogado	Integrante
J. Pablo Undurraga Fourcade	Médico	Integrante
Iris Delgado Becerra	Profesora, M.Sc. Bioestadística	Integrante

PCS/JMM
Nº Ref.:EC1707587/21

RESOLUCIÓN EXENTA RW N° 30574/21
Santiago, 17 de noviembre de 2021

VISTO ESTOS ANTECEDENTES: la presentación del Director Técnico Sra. Claudia Andrea Asenjo Lobos en representación de UNIVERSIDAD DEL DESARROLLO, la que solicita autorización para el uso provisional sin registro sanitario para el producto farmacéutico detallado en la parte resolutiva, para la realización del Estudio Clínico patrocinado por THE GEORGE INSTITUTE FOR GLOBAL HEALTH, a efectuarse en el centro CLINICA ALEMANA DE SANTIAGO y CLINICA ALEMANA DE SANTIAGO SEDE LA DEHESA, bajo la responsabilidad de la DRA. PAULA MUÑOZ VENTURELLI.

CONSIDERANDO:

· La opinión favorable del Comité Ético Científico Facultad de Medicina Clínica Alemana de Santiago - Universidad del Desarrollo, el cual se encuentra debidamente acreditado.
· El Informe Técnico Científico N° 104/21 de la Sección Estudios Clínicos.
· La constancia de cumplimiento de Buenas Prácticas de Manufactura (BPM) de la planta de fabricación, emitida por la autoridad sanitaria para el sitio de fabricación.
· Que ningún producto farmacéutico podrá ser distribuido en el país sin que haya sido registrado (art. 97° del Código Sanitario).

TENIENDO PRESENTE: las disposiciones contenidas en el artículo 96 y 99 de la Ley 20724 del 14 de febrero de 2014, que modifica el Código Sanitario, Decreto con Fuerza de Ley N° 725 de 1968, el Decreto Supremo N°3/10 , la Ley 20850 del 6/6 de 2015, del Ministerio de Salud; y los artículos 59º letra b) y 61º letra b) del DFL Nº 1 de 2005 y las facultades delegadas por la Resolución Exenta Nº 191 de 05 de febrero de 2021, del Instituto de Salud Pública de Chile, las Resoluciones N° 441/2012, 460/2015, 5161 y 5174 de 2016, y la Resolución N°403 del 2013 que aprueba la Norma Técnica N°151, dicto la siguiente:

R E S O L U C I Ó N

1.- **AUTORÍZASE** la ejecución del protocolo: "Un ensayo clínico internacional y pragmático, iniciado y conducido por investigador para determinar si 40 mg diarios de Atorvastatina pueden mejorar las funciones neurocognitivas en adultos con síntomas neurológicos prolongados producidos por el COVID", Protocolo N° NCT04904536, Fase Nº: III, de fecha 16 de septiembre de 2021 (versión 4.0), Formulario de Consentimiento Informado versión local 1.2, de fecha 22 de septiembre de 2021, sometidos a la evaluación ética del COMITÉ ÉTICO CIENTÍFICO FACULTAD DE MEDICINA CLINICA ALEMANA-UNIVERSIDAD DEL DESARROLLO, cuyo informe favorable se emitió con fecha 05 de octubre de 2021.

2.- El ensayo clínico se realizará cumpliendo los siguientes requisitos y condiciones:

a) El titular de esta autorización corresponde a UNIVERSIDAD DEL DESARROLLO.
b) El patrocinador ha presentado un documento legal de delegación de funciones en UNIVERSIDAD DEL DESARROLLO, con domicilio en Av. Plaza 680, LAS CONDES.
b) El Patrocinador THE GEORGE INSTITUTE FOR GLOBAL HEALTH ha presentado un documento legal de delegación de funciones en UNIVERSIDAD DEL DESARROLLO, con domicilio en Av. Plaza N° 680, Las Condes, Santiago.
c) El producto autorizado a usarse en dicho ensayo clínico es el que a continuación se detalla, en su concentración, forma farmacéutica, unidad de

medida, cantidad y presentación.

Ítem	Producto	Concentración/dosis unitaria	Forma farmacéutica	Unidad de medida	Cantidad	Presentación
1	Atorvastatina (Registro Sanitario F-19644/17)	40 mg	Comprimidos Recubiertos	Caja	2.200	Caja que contiene 30 comprimidos recubiertos
Fabricante: Laboratorio Chile S.A.						
Domicilios: Camino a Melipilla N° 9978, Maipú, Santiago, Chile.						

Almacenado a no más de 30 ºC, protegido de la luz, calor y humedad.

Procedencia: Droguería Pharmavisan (Ñuñoa, Santiago, Chile).

3.- El protocolo se llevará a cabo en los Centros: CLINICA ALEMANA DE SANTIAGO con domicilio en AVENIDA VITACURA N° 5951, VITACURA, SANTIAGO, Y CLINICA ALEMANA DE SANTIAGO SEDE LA DEHESA con domicilio en AV. JOSE ALCALDE DELANO - LA DEHESA N° 12205, LO BARNECHEA, SANTIAGO.

4.-UNIVERSIDAD DEL DESARROLLO, se responsabilizará de certificar la calidad del producto que utiliza.

5.- El producto será almacenado en la bodega de DROGUERÍA PHARMAVISAN S.A., ubicada en: Calle Los Jardines N° 261, ÑUÑOA, REGION METROPOLITANA, para su posterior distribución a los centros de estudio.

6.- El Titular tendrá las obligaciones siguientes:

- Informar al Instituto de Salud Pública de Chile las sospechas de eventos adversos serios a medicamentos acontecidos durante la ejecución del respectivo protocolo de estudio clínico.
- Remitir "Informes Periódicos de Seguridad de Productos Farmacéuticos usados en Investigación Clínica (IPSI; en ingles DSUR, Development Safety Update Report), entregando información de seguridad de un medicamento cuyo uso clínico se investiga y que, entre varios elementos, contiene información de eventos adversos acontecidos durante la ejecución del protocolo de investigación correspondiente, así como una evaluación científica al respecto.
- Cumplir en todo momento con las prescripciones del protocolo cuya ejecución se ha autorizado y con las Buenas Prácticas Clínicas; en lo que corresponda.
- Acceder a las inspecciones que puedan ser realizadas por la autoridad sanitaria y que están destinadas a la verificación del cumplimiento de la autorización y de las Buenas Prácticas Clínicas.
- Notificar cambios de orden administrativo relacionados con la ejecución del estudio clínico, y no incorporados en las menciones que más adelante se indican.
- Remitir Informe de cierre anticipado o, en su defecto, informe final al término del ensayo clínico. En el caso de los estudios que se realicen en más de un país, se deberá incluir además los antecedentes relacionados con la ejecución del estudio en el extranjero

7.- Esta resolución tendrá vigencia por un plazo de un año, desde la fecha de la presente resolución, previo el cuál deberá, en caso de que lo requiera, solicitar una renovación de autorización a este Instituto.

INSTITUTO DE SALUD PÚBLICA DE CHILE
AGENCIA NACIONAL DE MEDICAMENTOS
JEFE

Q.F. JUAN ROLDÁN SAELZER
JEFE (S) DEPARTAMENTO
AGENCIA NACIONAL DE MEDICAMENTOS
INSTITUTO DE SALUD PÚBLICA DE CHILE

DISTRIBUCIÓN
INTERESADO
ARCHIVO ANAMED

INSTITUTO DE SALUD PÚBLICA DE CHILE
MINISTRO DE FE

Transcrito Fielmente
Ministro de Fe
Carolina Corvalán Villaseca
