# Supplementary material for: STatin TReatment for COVID-19 to Optimise NeuroloGical recovERy (STRONGER): study protocol for a randomised, open label clinical trial in patients with persistent neurological symptoms after COVID-19 infection
Source: BMJ Open. 2025 Apr 14;15(4):e089382. doi: 10.1136/bmjopen-2024-089382 (PMC11997840; doi:10.1136/bmjopen-2024-089382)
Supplement: Supplementary file 3 [file bmjopen-2024-089382.reviewer_comments.pdf]

PEER REVIEW HISTORY

BMJ Open publishes all reviews undertaken for accepted manuscripts. Reviewers are asked to complete a checklist review form and are provided with free text boxes to elaborate on their assessment. These free text comments are reproduced below.

ARTICLE DETAILS

Title (Provisional)

STatin TReatment for COVID-19 to Optimise NeuroloGical recovERy (STRONGER): study protocol for a randomised, open label clinical trial in patients with persistent neurological symptoms after COVID-19 infection

Authors

Delfino, Carlos; Carcel, Cheryl; Lin, Xiaolei; Muñoz, Paula; Naismith, Sharon L; Woodward, Mark; Peters, Ruth; Wijesuriya, Nirupama; Law, Meng; Harding, Ian H; Wang, Xia; Elliott, Julian; Leder, Karin; Hutchings, Owen; Stecher, Ximena; Zoungas, Sophia; Anderson, Craig

---

VERSION 1 - REVIEW

**Reviewer**	**1**
**Name**	**Umakanthan, Srikanth**
**Affiliation**	**The University of the West Indies at St Augustine, Medical Sciences**
**Date**	**02-Oct-2024**
**COI**	**None**

Include reasons for selecting Atorvastatin in STRONGER.

“Open label” should be defined for multidisciplinary readers' purposes.

The authors have provided the reasons for neurocognitive problems encountered due to long COVID-19 exposure, but the features of this condition should be elaborated apart from the neuroimaging description provided in the manuscript.

Is there any input from the histopathologist in your study? As mentioned in the manuscript, the details of gliosis and neuropil are best evaluated by pathologists than as identified by radiology, which can be non-specific.

What formula is used to generate adequate sampling in your study?

Is there any role of bias in your study?> if yes how would that be eliminated?

Indicate the categorization of participants based on variables (dependable and independent)

A 20% dropout assumption is very high. Indicate the reasons for it and what measures you would take to limit this dropout estimate.

Statistical analysis should be provided in detail.

Are there any assumed limitations for this study? To be elaborated.

**Reviewer**	**2**
**Name**	**Prasad, Kameshwar**
**Affiliation**	**All India Institute of Medical Sciences**
**Date**	**03-Nov-2024**
**COI**	**None**

Comments on the paper entitled, '**STatin TReatment for COVID-19 to Optimise NeuroloGical recovERy (STRONGER): study protocol for a randomised, open label clinical trial in patients with persistent neurological symptoms after COVID-19 infection**

Major points

Primary analysis is planned using logistic regression models. How will the dependent variable be dichotomised. Needs to be prespecified to avoid data-driven dichotomisation.

According to ICEMAN criteria for credible subgroup analyses, it is important not only to specify the subgrouping variable, but also the anticipated direction of effect across different levels of the subgroups according to their value labels.

Clinically meaningful score on SDMT is based on a paper with patients of multiple sclerosis. Whether it can be extrapolated to patients with long covid is a moot point. Authors need to discuss this in more detail.

Minor points

Line 40. Prospective word is uncalled for. Randomised trials have to be prospective, can't be retrospective or cross-sectional.

Multiple places: O in PROBE stands for 'Open-label'

Line 139: The phrase 'a severe co-morbid medical or psychiatric condition that prevents participation' is vague and likely to introduce avoidable heterogeneity in the sample.

Line 161: cognitive tests: provide a list to be clear.

Line 162: Before randomisation, 'After consent'

Line 165: 'focuses' instead of 'focus'

Line 169: What does specialised mean? Will they be clinical psychologists?

Line 182: 'documents' instead of 'document'

Line 187: 'documents' instead of 'document'

Figure 1 may be modified to show the point where consent will be taken.

VERSION 1 - AUTHOR RESPONSE

Reviewer: 1

1. **Include reasons for selecting atorvastatin in STRONGER.**

Response: We selected atorvastatin for the STRONGER study because of its high lipophilicity, which allows it to cross the blood-brain barrier; this was mentioned in the Introduction (lines 121-126). This characteristic makes atorvastatin a promising candidate to address the neurological symptoms associated with long COVID-19, as it has the potential to modulate the neurovascular unit and reduce acute inflammation.

2. **"Open label" should be defined for multidisciplinary readers' purposes.**

Response: To ensure clarity for a multidisciplinary audience, we have added a definition of 'open label' in the manuscript (lines 140-141).

3. **The authors have provided the reasons for neurocognitive problems encountered due to long COVID-19 exposure, but the features of this condition should be elaborated apart from the neuroimaging description provided in the manuscript.**

Response: We have incorporated a more detailed description of the neurocognitive characteristics associated with long COVID and reorganized the information in the Introduction to provide a more comprehensive understanding of the condition (lines 94-100).

4. **Is there any input from the histopathologist in your study? As mentioned in the manuscript, the details of gliosis and neuropil are best evaluated by pathologists than as identified by radiology, which can be non-specific.**

Response: We agree that histopathological analysis would provide more specific evaluation of gliosis and neuropil changes. However, the current study does not include any histopathological assessment; rather, only non-invasive methods are used, in particular that of advanced MRI techniques which offer an approximation of pathological processes. These imaging methods are well-suited for evaluating neuroinflammatory and structural changes in vivo, and provide a useful biomarker substudy. We have clarified this approach in the manuscript (lines 97-104).

5. **What formula is used to generate adequate sampling in your study?**

Response: The study statistician used the software package PASS version 15 (NCSS, Kaysville, UT; http://www.ncss.com/software/pass). (lines 221-222)

6. **Is there any role of bias in your study? if yes how would that be eliminated?**

Response: As this is a clinical trial, a major source of bias is (i) selection, so we plan to compare the characteristics of participants with those in published cohorts, and between those who were screened. As this is an open trial, another main source of bias relates to (ii) differential drop-out, for example in the control 'no treatment' group failing to return to follow-up assessments or withdrawing from the study. We will try to reduce this by ensuring all participants fully understand the importance of participation and follow-up irrespective of ceasing the study medication, and in maintaining good communication and relationships with participants. A final source of bias is (iii) behavioural –

participants in either group may change their lifestyle and attitude simply due to participation or in response to factors outside of the trial. We will monitor this by asking questions about use of concomitant medications, diet, smoking and exercise, and through use of actigraphy as a proxy measure of sleep and physical activity. There is low likelihood that the control 'no treatment' group will receive atorvastatin (i.e. treatment crossover) as this can only be prescribed for people who are at high-risk of cardiovascular disease. (lines 297-310)

7. **Indicate the categorization of participants based on variables (dependable and independent)**

Response: For the primary outcome SDMT, the main analysis does not categorize participants, but instead analyze it as a continuous variable using linear regression. However, as a sensitivity analysis, SDMT will be dichotomized according to population norms (an average score of 54 for those aged 44 years in a large national representative sample of Australians;[1] a median of 53.2 for those aged 43-47 year in Chile[2]), such that participants with will be categorised as having high or low SDMT scores

For secondary outcomes, PHQ-9 will be dicotamised into depression (⩾10) and no depression (<10), PSQI dichotamised into poor (PSQI >5) and good (PSQI ⩽5) sleep quality, GAD-7 dichotamised as generalised (⩽10) and non-generalised (>10), anxiety or categorised into mild (⩽5), moderate (6 to 9) and severe (⩾10) anxiety.

The independent variable is binary group indicator based on the treatment allocation that follows the ITT principle.

8. **A 20% dropout assumption is very high. Indicate the reasons for it and what measures you would take to limit this dropout estimate.**

Response: We agree this is high but patients with long neurological COVID tend to be young or middle aged females who have considerable anxiety and help seeking behaviour associated with the unusual nature of the symptoms, concerns over 'inflammation in the brain', lack of appropriate specialist referral pathways and absence of proven treatments. We have found that a baseline assessment of a participant takes 2-3 hours.

9. **Statistical analysis should be provided in detail.**

Response: We have provided more details of the statistical analysis plan in this subsection of the manuscript, describing strategies that will be used to present baseline characteristics, primary and secondary endpoints, subgroup analysis, and safety outcomes (lines 235-270). A detailed statistical analysis plan (SAP) will be drafted with step-by-step instructions on the main and sensitivity analysis of the primary and secondary endpoints. This will be completed prior to completion of the follow-up of participants and unblinding of the data.

10. **Are there any assumed limitations for this study? To be elaborated**.

Response: Thanks for this comment. The limitations of the study are incorporated into the Conclusion section of the manuscript (lines 338–342). Additionally, they are highlighted in the 'Strengths and Limitations' section at the beginning of the manuscript for clarity (lines 62-69).

References

1. *Kiely KM, Butterworth P, Watson N, Wooden M. The Symbol Digit Modalities Test: Normative data from a large nationally representative sample of Australians. Arch Clin Neuropsychol. 2014 Dec;29(8):767-75*

2. *Arango-Lasprilla JC, Rivera D, Rodríguez G, Garza MT, Galarza-Del-Angel J, Rodríguez W, Velázquez-Cardoso J, Aguayo A, Schebela S, Weil C, Longoni M, Aliaga A, Ocampo-Barba N, Saracho CP, Panyavin I, Esenarro L, Martínez C, García de la Cadena C, Perrin PB. Symbol Digit Modalities Test: Normative data for the Latin American Spanish speaking adult population. NeuroRehabilitation. 2015;37(4):625-38*

Reviewer: 2

Major points

1. **Primary analysis is planned using logistic regression models. How will the dependent variable be dichotomised. Needs to be prespecified to avoid data-driven dichotomisation.**

Response: Thanks for the comment. As outlined previously, we have modified the statistical analysis plan, with the main analysis of the primary outcome SDMT conducted using linear regression (not as first stated in the protocol). SDMT will be included as the dependent variable, treatment allocation as the independent variable, and age, sex and study site included as the covariates. As a sensitivity analysis, we will dichotomize the primary outcome into high and low SDMT based population norms for participants aged 44 years old (mean age in this trial). The dichotomized SDMT will be analyzed using logistic regression, where high and low SDMT included as the dependent variable, treatment allocation as the independent variable, and age, sex and study site included as the covariates. The cut off values for dichotomising the SDMT is based on evidence and is not data driven.

2. **According to ICEMAN criteria for credible subgroup analyses, it is important not only to specify the subgrouping variable, but also the anticipated direction of effect across different levels of the subgroups according to their value labels.**

Response: This is a useful comment. We prespecified 5 subgroups for analysis:

- age (age <60 vs. ⩾60 years);
- time since COVID-19 diagnosis (time < 6 months vs. ⩾ 6 months)
- baseline CRP levels (CRP < 5 mg/L, 5-9 mg/ L, ⩾10 mg/ L);
- ethnicity (White, Hispanics, Other)
- prior cardiovascular risk (prior history of hypertension, diabetes, smoker, BMI > 30)

Based on clinical knowledge, we hypothesize that a better treatment effect is anticipated in those with inflammation (high baseline CRP level, shorter time since COVID-19 diagnosis), and participants who are young, non-White, and with elevated cardiovascular risk. (lines 257-270).

3. **Clinically meaningful score on SDMT is based on a paper with patients of multiple sclerosis. Whether it can be extrapolated to patients with long covid is a moot point. Authors need to discuss this in more detail**.

Response: We consider multiple sclerosis is a good surrogate disease for long COVID as it preferentially affects younger female adults and involves diffuse inflammation of the cerebral white matter to produce patchy impairment of executive cognitive function. We acknowledge that a clinically meaningful score on the SDMT may be different in long neurological COVID, but have updated the manuscript with a reference to a recent systematic review on neurocognitive impairment in long COVID. We believe this finding underscores the use of neuropsychological tests, such as the SDMT, to evaluate cognitive deficits in our study population (https://doi.org/10.1093/arclin/acae042).

Minor points

4. **Line 40. Prospective word is uncalled for. Randomised trials have to be prospective, can't be retrospective or cross-sectional.**

Response: We acknowledge that the term 'prospective' is redundant in this context. We have removed the word accordingly to improve the clarity and precision of the manuscript

5. **Multiple places: O in PROBE stands for 'Open-label'**

Response: We have corrected the definition of PROBE to ensure that 'O' is accurately stated as 'Open-label' throughout the manuscript.

6. **Line 139: The phrase 'a severe co-morbid medical or psychiatric condition that prevents participation' is vague and likely to introduce avoidable heterogeneity in the sample.**

Response. We agree that this phrase may be perceived as vague and lead to unintended heterogeneity. We have revised the wording with a clearer definition, specifying examples of severe comorbidities that would prevent participation, such as renal failure or schizophrenia (line 158). This clarification aims to reduce variability and enhance the consistency of the sample criteria.

7. **Line 161: cognitive tests: provide a list to be clear.**

Response: To enhance clarity and considering that a large number of cognitive tests are involved, we have simply listed them in Table 1. This should make the information more accessible to readers.

8. **Line 162: Before randomisation, 'After consent'**

Response: The consent process is conducted during the preliminary visit (line 177). To enhance clarity, this information is also added to Figure 1.

9. **Line 165: 'focuses' instead of 'focus'**

Response: We have corrected the text to use 'focuses' instead of 'focus' to ensure grammatical accuracy (new line 185)

10. **Line 169: What does specialised mean? Will they be clinical psychologists?**

Response: 'Specialised' refers to psychologists with specific training and experience in administering cognitive assessments in research settings. While they may not necessarily be a clinical psychologist, they will have the requisite expertise to ensure the validity and reliability of the assessments. We have clarified this in the text to avoid ambiguity (lines 198-200).

11. **Line 182 and 187: 'documents' instead of 'document'**

Response: We have updated the text to use 'documents' instead of 'document' in both instances to ensure grammatical consistency.

12. **Figure 1 may be modified to show the point where consent will be taken.**

Response: We have updated Figure 1 to clearly indicate the point at which consent is obtained, ensuring alignment with the study procedures outlined in the manuscript.

VERSION 2 - REVIEW

Reviewer 1

Name **Umakanthan, Srikanth**

Affiliation **The University of the West Indies at St Augustine, Medical Sciences**

Date **24-Feb-2025**

COI

Comments for revision are suitable and reasoned as necessary.
